# Hair-Derived Exposome Exploration of Cardiometabolic Health: Piloting a Bayesian Multitrait Variable Selection Approach

**DOI:** 10.1021/acs.est.3c08739

**Published:** 2024-03-13

**Authors:** Rin Wada, Feng-Jiao Peng, Chia-An Lin, Roel Vermeulen, Alba Iglesias-González, Paul Palazzi, Barbara Bodinier, Sylvie Streel, Michèle Guillaume, Dragana Vuckovic, Sonia Dagnino, Julien Chiquet, Brice M. R. Appenzeller, Marc Chadeau-Hyam

**Affiliations:** † Department of Epidemiology and Biostatistics, School of Public Health Imperial College London, London W2 1PG, U.K.; ‡ MRC Centre for Environment and Health Imperial College London, London W2 1PG, U.K.; § Human Biomonitoring Research Unit, Department of Precision Health, Luxembourg Institute of Health, Strassen L-1445, Luxembourg; ∥ Institute for Risk Assessment Sciences, Utrecht University, Utrecht 3584 CM, The Netherlands; ⊥ Department of Public Health Sciences, University of Liege, Liege 4000, Belgium; # Transporters in Imaging and Radiotherapy in Oncology (TIRO), Institut des sciences du vivant Fréderic Joliot, CEA, Université Côte d’Azur, Nice 06107, France; ¶ Université Paris-Saclay, AgroParisTech, INRAE, UMR MIA Paris-Saclay, Palaiseau 91120, France

**Keywords:** exposome, cardiometabolic health, environmental epidemiology, hair analysis, pollutants, multitrait analysis

## Abstract

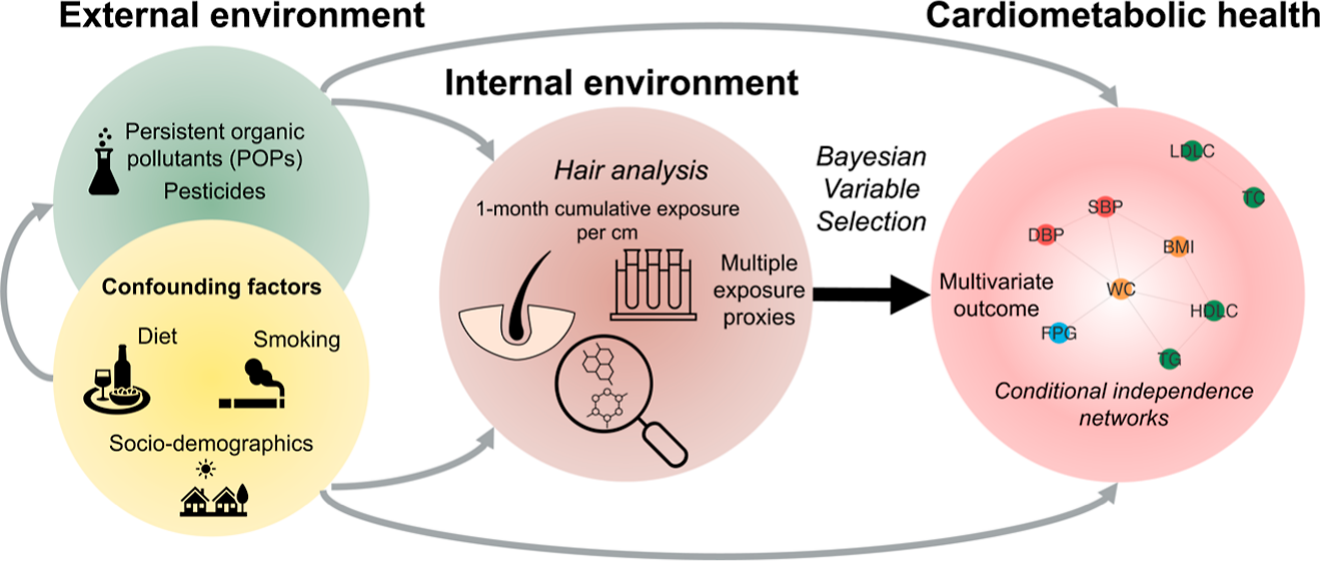

Cardiometabolic health is complex and characterized by an ensemble of correlated and/or co-occurring conditions including obesity, dyslipidemia, hypertension, and diabetes mellitus. It is affected by social, lifestyle, and environmental factors, which in-turn exhibit complex correlation patterns. To account for the complexity of (i) exposure profiles and (ii) health outcomes, we propose to use a multitrait Bayesian variable selection approach and identify a sparse set of exposures jointly explanatory of the complex cardiometabolic health status. Using data from a subset (*N* = 941 participants) of the nutrition, environment, and cardiovascular health (NESCAV) study, we evaluated the link between measurements of the cumulative exposure to (*N* = 33) pollutants derived from hair and cardiometabolic health as proxied by up to nine measured traits. Our multitrait analysis showed increased statistical power, compared to single-trait analyses, to detect subtle contributions of exposures to a set of clinical phenotypes, while providing parsimonious results with improved interpretability. We identified six exposures that were jointly explanatory of cardiometabolic health as modeled by six complementary traits, of which, we identified strong associations between hexachlorobenzene and trifluralin exposure and adverse cardiometabolic health, including traits of obesity, dyslipidemia, and hypertension. This supports the use of this type of approach for the joint modeling, in an exposome context, of correlated exposures in relation to complex and multifaceted outcomes.

## Abstract 2

We propose, as a proof-of-concept, a multitrait Bayesian variable selection approach to model the joint effects of hair-derived cumulative exposure to (*N* = 33) pollutants on complex and multivariate cardiometabolic health.

## Introduction

The exposome concept refers to the totality of non genetic factors an individual is exposed to during the life-course and reaches the internal environment. This solicits multiple adaptive responses, hence leaving biological imprints, which may accumulate over the life-course.^1,2^ Assessing how multiple external exposures may jointly generate a specific biological response, which in-turn may affect health outcomes, is crucial to better understand the associations between complex exposures and health and to hypothesize possible mechanisms at stake.^3^

Hair has emerged as an effective biomonitoring tool for the measurement of trace elements embodied from multiple environmental exposures. This overcomes the limitations of conventional matrices, such as blood and urine, where short half-life of chemicals and their metabolites may lead to increased variability and time-varying measurements, and its suitability for exposure assessment has been previously discussed.^4−6^ Hair-derived measurements robustly capture the cumulative and average long-term exposure history, offering a stable reflection of individual-level exposures, with preparatory techniques effectively removing interference from external chemicals and extraction methods enhancing trace element recovery.^7^ For example, hair analysis has been used successfully to evaluate the exposure to endocrine disrupting chemicals and metals.^8−11^ Given its low expenses for sampling and the ability to trace comprehensive exposure history, the hair exposome has been applied to evaluate the effects of environmental factors on various areas of health, including reproductive health.^12^ The development of advanced statistical methods is required to appropriately analyze these hair-derived measurements and to conduct studies from an exposome perspective.

Cardiovascular disease (CVD) is a group of disorders affecting the heart and blood vessels representing a leading cause of morbidity and mortality in most European populations.^13^ The main behavioral risk factors of CVD are unhealthy diet, physical inactivity, tobacco use, and misuse of alcohol, which may affect cardiometabolic health and induce obesity, hypertension, dyslipidemia, and diabetes mellitus. The multifactorial etiology of CVD warrants a holistic approach to consider the effect of multiple exposures on cardiometabolic health as proxied by a multivariate set of clinical phenotypes.

Environmental pollutants have been linked with both clinical risk factors for CVD development and the risk and severity of CVD itself.^14,15^ These pollutants, often originating from industrial activities, vehicular emissions, and agricultural practices, can infiltrate the air, water, and soil, thereby entering the body through various routes and potentially affecting cardiometabolic health due to their toxicity and endocrine-disrupting potential. While there is substantial evidence linking air pollutants and heavy metals to the development and exacerbation of cardiovascular conditions,^16,17^ the role of exposure to persistent organic pollutants (POPs) and pesticides in the pathogenesis of CVD is less established.^18−22^

In this context, we propose to adopt an exposome approach to assess the association between the environmental pollutants, as measured by multiple pollutant levels in hair, and cardiometabolic health. The nutrition, environment and cardiovascular health (NESCAV) study is a population-based study initiated with the aim of standardizing instruments to evaluate cardiometabolic health and to identify the potential gaps in the current inter-regional CVD prevention across the Greater Region, located in the center of Europe.^23^ Hair specimens from the study participants were analyzed to generate a panel of 76 compounds. These included 33 POPs, pesticides, and their metabolites, which provide a holistic view of environmental pollutants the participants were subjected to and how these were embodied. We investigate how, jointly, these exposure proxies are associated with cardiometabolic health. Unlike previous studies where cardiometabolic health had been modeled as a single outcome, such as the presence of metabolic syndrome,^24,25^ we propose here to use a multivariate outcome model whereby cardiometabolic health is characterized by several traits related to obesity, dyslipidemia, hypertension, and insulin resistance phenotypes that, together, capture the complexity of cardiometabolic health. We use a Bayesian variable selection (BVS) approach, accommodating multivariate outcomes, to identify sparse sets of exposures that jointly predict an ensemble of phenotypes. The data set used in the present study has been fully described elsewhere.^23,26−31^

## Materials and Methods

### Study Population

The NESCAV study is an inter-regional population-based study across the Greater Region (Grand-Duchy of Luxembourg, Wallonia in Belgium and Lorraine in France) conducted between 2007 and 2013, focusing on the influence of social factors, lifestyle behaviors, and environmental exposures on cardiometabolic health.^23^ A total of 3,006 participants aged 18–69 years were recruited with informed consent. A cross-sectional survey of the demographic, social, behavioral, and environmental factors was performed via in-person interviews, self-administered questionnaires, and clinical examinations, including the sampling of blood and hair. Of these, 1,428 participants were considered for the present study after excluding participants with insufficient amounts of hair sampled or missing/invalid clinical measurements and participants from Lorraine in France since only 75 had a sufficient amount of hair.

To assess cardiometabolic health, the present study considered the clinical measurements of phenotypic traits that define the four main cardiovascular domains: (i) obesity [body mass index (BMI) and waist circumference (WC)], (ii) dyslipidemia [serum levels of triglycerides (TG), total cholesterol (TC), high-density lipoprotein cholesterol (HDL-C), and low-density lipoprotein cholesterol (LDL-C)], (iii) hypertension [systolic blood pressure (SBP) and diastolic blood pressure (DBP)], and (iv) diabetes mellitus [fasting plasma glucose levels (FPG)]. The methods of the clinical survey have been previously described in detail.^26^

Age, gender, and educational attainment were recorded for each participant. Educational attainment was measured by the highest level of qualification attained in three categories: low (below high school), intermediate (high school), and high (above high school). Lifestyle behaviors including dietary habits, smoking status, physical activity, and alcohol consumption were assessed via questionnaire. The method of the semi-quantitative food frequency questionnaire has been described previously.^28^ The results from this questionnaire generated five numeric variables: (i) energy (kcal/day), (ii) proteins (g/day), (iii) fats (g/day), (iv) carbohydrates (g/day), and (v) fibers (g/day). These were summarized using principal component analysis (PCA), and we retained as many components as needed to explain more than 95% of the variance of the five original variables. The smoking status was categorized as never, former, and current smoker. Physical activity was categorized as active, moderately active, and inactive according to the International Physical Activity Questionnaire (IPAQ) scoring criteria.^27^ Alcohol consumption was coded in three categories: low (<0.1 g/day), moderate (0.1–10 g/day), and high (>10 g/day).

The embodiment of environmental pollution was assessed by measuring a panel of exogenous chemicals and their metabolites from hair specimens collected from the scalp in the posterior vertex region of the head. This panel included 13 POPs, which consisted of three polychlorinated biphenyls (PCBs), one polybrominated diphenyl ether, and nine organochlorine pesticides. Additionally, 20 nonpersistent pesticides were measured, including 9 organophosphorus pesticides, 4 pyrethroids, 2 phenylpyrazoles, 2 carbamates, 1 carboxamide, 1 dinitroaniline, and 1 oxadiazole. Based on the average growth rate of hair, every centimeter of the sample represents one month of cumulative exposure.^32^ Thus, the concentration (pg/mg) of each compound measured in hair samples was used as a proxy for the cumulative exposure from four months before the assessment for a given individual. All hair samples were analyzed at the Human Biomonitoring Research Unit of Luxembourg Institute of Health. The methods for hair sampling, chemical analysis, including quality control, have been previously detailed.^6,8,30,31,33^

### Statistical Analysis

All analyses were performed in R, version 3.6.3. The hair-derived measurements of 33 chemical compounds (POPs, pesticides, and their metabolites) were considered in the study. A total of 941 participants were included in the analyses after excluding participants with missing chemical measurements (*N* = 439) and those missing ≥50% of the dietary intake information (*N* = 48). All compounds were detected in ≥10% of the samples, with the proportion of samples presenting concentrations above the limit of detection (LOD) presented in Table S2. Concentrations below the LOD were imputed by sampling a truncated Gaussian distribution between zero and the LOD for each compound. Missing measurements (arising from technical issues during hair analysis, <50% per compound) and missing questionnaire information were imputed using the random forest algorithm implemented in the R package *missForest*.^34^ The chemical measurements were then transformed to the log_10_ scale.

#### Descriptive Analysis

Descriptive analysis was performed to identify the sets of health outcomes and blocks of correlated exposures to be modeled jointly. Within the (*N* = 9) measured cardiometabolic traits, we sought for those that correlated and/or co-occurred through a network of pairwise partial correlations (conditional independence network) using a stability-enhanced graphical LASSO modeling approach,^35^ where we optimize a score measuring the overall stability of the model to jointly calibrate two hyperparamters: (i) the penalty parameter λ controlling the amount of shrinkage and (ii) the threshold in selection proportion π above which the corresponding edge is considered stable.^36^ To quantify model stability, the edges were partitioned into three categories (stably selected, stably excluded, or unstably selected) based on their selection counts calculated over the graphical LASSO models fitted on 100 subsamples of 50% of the data (for a given pair of λ and π). By considering the selection counts as independent observations, the stability score was derived based on the likelihood of observing this classification under the hypothesis of instability (where all edges share the same probability of being selected, hence yielding a uniform distribution of the selection proportion). To identify blocks of correlated exposures to be considered jointly as predictors, we combined dietary intake information and chemical measurements and estimated a multiblock conditional independence network. This relied on the same approach, but the two hyperparameters were calibrated by using a block-specific stability score to account for the block correlation structure of exposures. We introduced error control in the network calibration and set an upper bound for the expected number of falsely selected edges, or Per-Family Error Rate, to 10 and 20 for the outcome and exposure networks, respectively.

Communities within the inferred exposure network were detected using the Louvain method, an algorithm that groups densely connected nodes together based on the optimization of the modularity metric with equal weights assigned to edges.^37^

As an exploratory approach, a series of linear regression models (33 × 9 models) was run relating each trait against hair-derived measurements of each exposure. Models were adjusted for age, gender, educational attainment, smoking status, and dietary intake PCA scores. Statistical significance was assessed based on a Bonferroni-corrected significance level controlling the family wise error rate below 0.05 (per-test significance level of <0.05/33).

#### Bayesian Variable Selection

GUESS is a computationally optimized BVS algorithm combining an evolutionary stochastic search Monte Carlo Markov Chain (MCMC) algorithm with parallel tempering, accommodating multiple continuous phenotypes by modeling the covariance structure among response variables.^38,39^ The algorithm is available in the R package *R2GUESS* v2.0.^39,40^ With the hair-derived measurements as predictors, GUESS was run to select variables jointly explanatory of (i) each trait separately and (ii) selected complementary traits, identified in the conditional independence network, serving as a multivariate outcome. All models were adjusted for age, gender, educational attainment, smoking status, and dietary intake PCA scores. Sparsity was imposed by setting the a priori expected model size (number of true associations) and its standard deviation to *E* = 5 and *S* = 4, and the truncation parameter was set to *F* = 7. The prior model size was 99% likely to range from 0 to 14 with a maximum model size of *T* = 33 (Table S3). GUESS was run for 30,000 sweeps with 10,000 sweeps as burn-in, with three chains that run in parallel. The validity of these parameters was evaluated by inspecting the traces of the model to ensure that convergence had been achieved. The average computational times for the single- and multitrait analyses were 8 and 20 h when performed on a HPC cluster computer with 1Gb of RAM using the Imperial College Research Computing Service (DOI: 10.14469/hpc/2232). An overview of the main preprocessing steps and analyses conducted is presented in Supplementary Figure S1.

#### Postprocessing of the GUESS Output

R2GUESS outputs a list of all visited models. The log-conditional posterior of a given model measures the quality of the fit of the model to the data and is scale-free. It is affected by the model complexity, and to ensure comparability across models, the posterior Model Posterior Probability (MPP) can be calculated instead based on the number of times a given model has been visited across all visited models. MPP is a proxy for individual model importance among all visited models throughout the MCMC run. The per-feature marginal contribution to the set of visited models is measured by the Marginal Posterior Probability of Inclusion (MPPI), which is a per-feature (model importance) weighted frequency of inclusion across all models visited. The MPPI can be interpreted as the posterior strength of association of a given predictor and the outcome(s). These outputs are previously described in detail.^38,41^ To identify statistically significant features jointly contributing to outcome prediction, we defined a threshold in MPPI, which controlled the empirical false discovery rate (FDR) to be below 0.05 through a permutation procedure, as previously proposed.^40^

As originally defined,^42^ Bayes Factor (BF) is the ratio of marginal probabilities of two different models and evaluates which of these two models is mostly supported by the data, and this could be interpreted as the Bayesian equivalent of a likelihood ratio test comparing two competing models in a frequentist framework. The BF comparing a model with and without a variable of interest then measures the contribution of that specific variable in the prediction of the outcome(s), and it can here be expressed as a function of the MPPI for that variable.^39^ The ratio of Bayes factor (RBF), for a given predictor and a given (set of) outcome(s), is then defined as the ratio of the BF measuring the importance of that variable and the BF for any variable found significantly associated with the outcome at a set empirical FDR level. The latter is calculated as the BF for a variable with the MPPI set to the MPPI threshold at a specified FDR level. The RBF, therefore, measures how the importance of a given variable is compared to that of any variable that would be called significant at a set FDR level, and it has been previously shown to enable the comparison of relative feature importance across different models.^39^ In practice, features with RBF ≥ 1 are to be interpreted as informative and as significantly contributing to the model performances.

Finally, the posterior distribution of the regression coefficients (effect size) for each selected variables in the top Best Models Visited (BMV) was estimated by simulating regression coefficient matrices (*N*_Σ_ × *N*_B_ = 2 × 2), the steps of which are previously outlined.^40^

#### Sensitivity and Attenuation Analysis

To assess the sensitivity to the way cardiometabolic health is measured (i.e., which physiological parameters to include in the multitrait analysis), we ran our multitrait analyses using different combinations of traits as outcomes.

To evaluate the role of potential confounders in our analyses, we performed a series of sensitivity analyses by running our BVS without adjustment and sequentially adjusting for (i) age, sex, (ii) educational attainment and smoking status, and (iii) dietary factors as summarized by the PCA scores of the three first PC scores (corresponding to our main model). We investigated the effect that the adjustment had on the per-feature MPPI.

## Results and Discussion

### Descriptive Analysis

The characteristics of the (*N* = 941) participants included in the study are summarized in Table 1. The ratio of females to males was high (69%), especially in Belgium with 75.3% being female. Differential patterns of educational attainment was observed between the two centers, with a higher proportion of those with low educational attainment in Luxembourg compared to participants from Belgium. The distribution of never, former, and current smokers was similar across the two centers, with never smokers being the most prevalent among all participants (53.8%).

**Table 1:** Overview of Population Characteristics Stratified by the Assessment Center^a^

	total	Belgium	Luxembourg
	*N* = 941	*N* = 481	*N* = 460
age (years), mean (SD)	44.8 (13.5)	44.6 (13.9)	44.9 (13.1)
Gender, *N* (%)			
Male	292 (31.0%)	119 (24.7%)	173 (37.6%)
Female	649 (69.0%)	362 (75.3%)	287 (62.4%)
Educational attainment, *N* (%)			
low	364 (38.7%)	120 (24.9%)	244 (53.0%)
intermediate	213 (22.6%)	120 (24.9%)	93 (20.2%)
high	358 (38.0%)	241 (50.1%)	117 (25.4%)
missing, *N* (%)	6 (0.6%)	0 (0.0%)	6 (1.3%)
Smoking status, *N* (%)			
never smoker	520 (55.3%)	257 (53.4%)	263 (57.2%)
former smoker	221 (23.5%)	105 (21.8%)	116 (25.2%)
current smoker	200 (21.3%)	119 (24.7%)	81 (17.6%)
energy (kcal/day), mean (SD)	2266.5 (763.3)	2214.1 (702.4)	2320.7 (818.9)
missing, *N* (%)	7 (0.7%)	6 (1.2%)	1 (0.2%)
proteins (g/day), mean (SD)	88.6 (30.7)	86.9 (27.2)	90.3 (33.9)
missing, *N* (%)	6 (0.6%)	3 (0.6%)	3 (0.7%)
fats (g/day), mean (SD)	96.6 (37.7)	92.4 (33.2)	100.8 (41.5)
missing, *N* (%)	9 (1.0%)	7 (1.5%)	2 (0.4%)
carbohydrates (g/day), mean (SD)	239.4 (89.0)	235.7 (82.3)	243.2 (95.5)
missing, *N* (%)	8 (0.9%)	6 (1.2%)	2 (0.4%)
fibres (g/day), mean (SD)	24.0 (8.9)	23.6 (7.8)	24.4 (9.8)
missing, *N* (%)	8 (0.9%)	6 (1.2%)	2 (0.4%)
Physical activity, *N* (%)			
inactive	196 (20.8%)	121 (25.2%)	75 (16.3%)
moderately active	288 (30.6%)	157 (32.6%)	131 (28.5%)
active	367 (39.0%)	129 (26.8%)	238 (51.7%)
missing, *N* (%)	90 (9.6%)	74 (15.4%)	16 (3.5%)
Alcohol consumption, *N* (%)			
low	175 (18.6%)	93 (19.3%)	82 (17.8%)
moderate	499 (53.0%)	233 (48.4%)	266 (57.8%)
high	260 (27.6%)	153 (31.8%)	107 (23.3%)
missing, *N* (%)	7 (0.7%)	2 (0.4%)	5 (1.1%)

a The proportion of missing values are shown for variables with missing values. SD = standard deviation.

**Figure 1:**
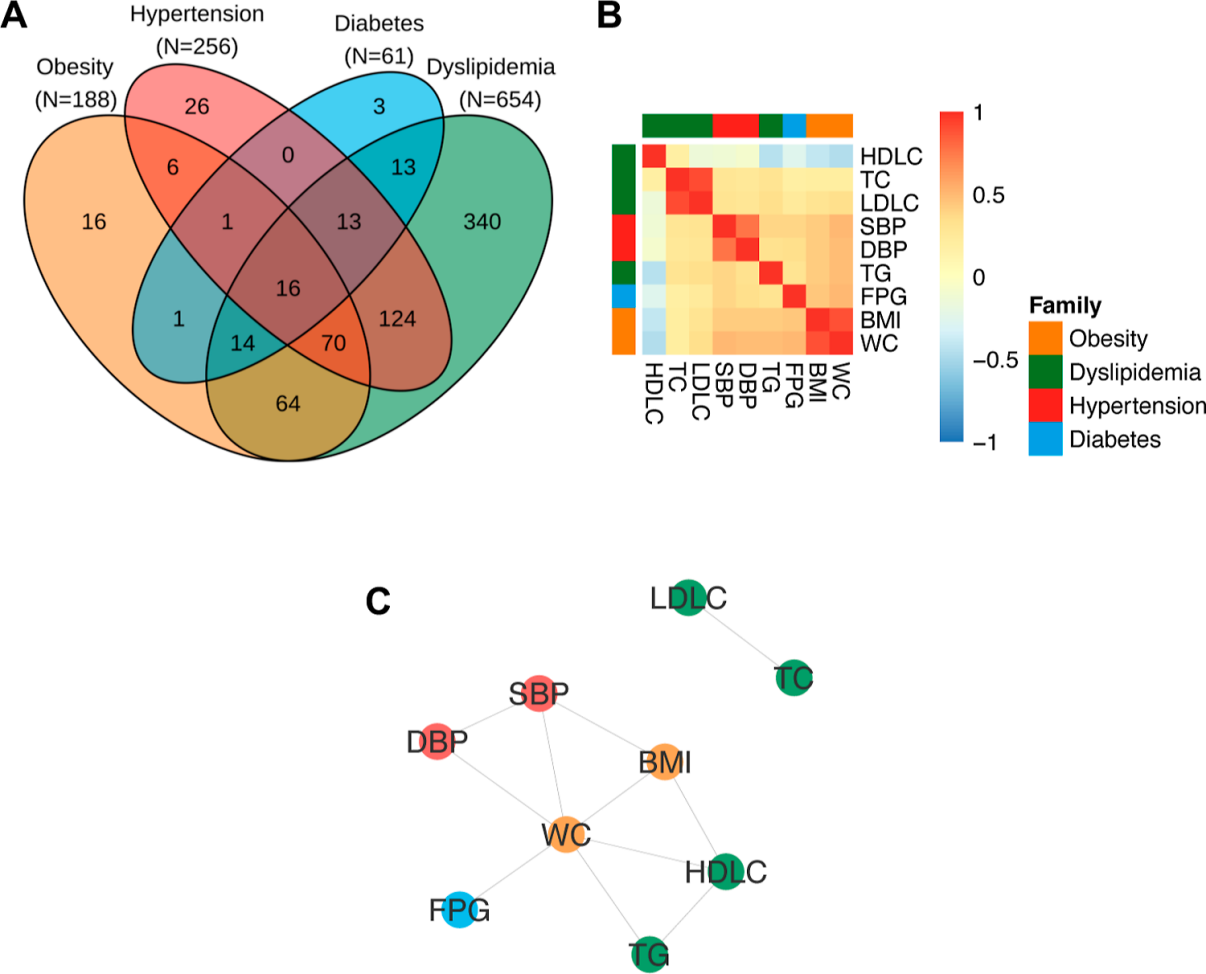
Descriptive summary of cardiometabolic health in the 941 study participants. Venn diagram representing comorbidity of the main cardiovascular outcomes (A); heatmap representing pairwise Spearman’s correlation coefficient between (*N* = 9) measured cardiometabolic traits (B) and the corresponding conditional independence network estimated using stability-enhanced graphical LASSO (C). Abbreviations: body mass index (BMI); waist circumference (WC); triglyceride (TG); total cholesterol (TC); high-density lipoprotein cholesterol (HDLC); low-density lipoprotein cholesterol (LDLC); systolic blood pressure (SBP); diastolic blood pressure (DBP); and fasting plasma glucose (FPG).

The prevalence of cardiometabolic conditions in the studied regions are among the highest in Europe, which warranted the need for the present study.^26^ Summary statistics of the nine measured cardiometabolic traits overall and by the center are summarized (Table S4), showing similar prevalence in both centers except for obesity, which was more prevalent in participants from Luxembourg. In the full study population, comorbidity was observed among the four cardiometabolic conditions (obesity, dyslipidemia, hypertension, and diabetes mellitus), with 86 participants having a combination of obesity, hypertension, and dyslipidemia (Figure 1A). This highlighted the need to consider multiple clinical phenotypes to grasp the complexity of cardiometabolic health. The pairwise correlation between BMI and WC and between LDL-C and TC was very high, with a Spearman’s correlation coefficient (ρ) of 0.89 and 0.90, respectively (Figure 1B). The pairwise correlation between SBP and DBP and between WC and FPG was moderately high (ρ = 0.76 and ρ = 0.51, respectively). The conditional independence network across all the measured cardiometabolic traits was calibrated via stability (Figure S2), and we identified six traits (BMI, WC, TG, HDL-C, SBP, and DBP) that were central to the network with each trait connected to at least two other traits (Figure 1C). By construction, the correlation between these six variables cannot be fully explained by the correlation with the other variables. We therefore hypothesize that these represent a set of complementary phenotypes capturing the complexity of cardiometabolic health, and we model them jointly in our multitrait analysis.

**Figure 2:**
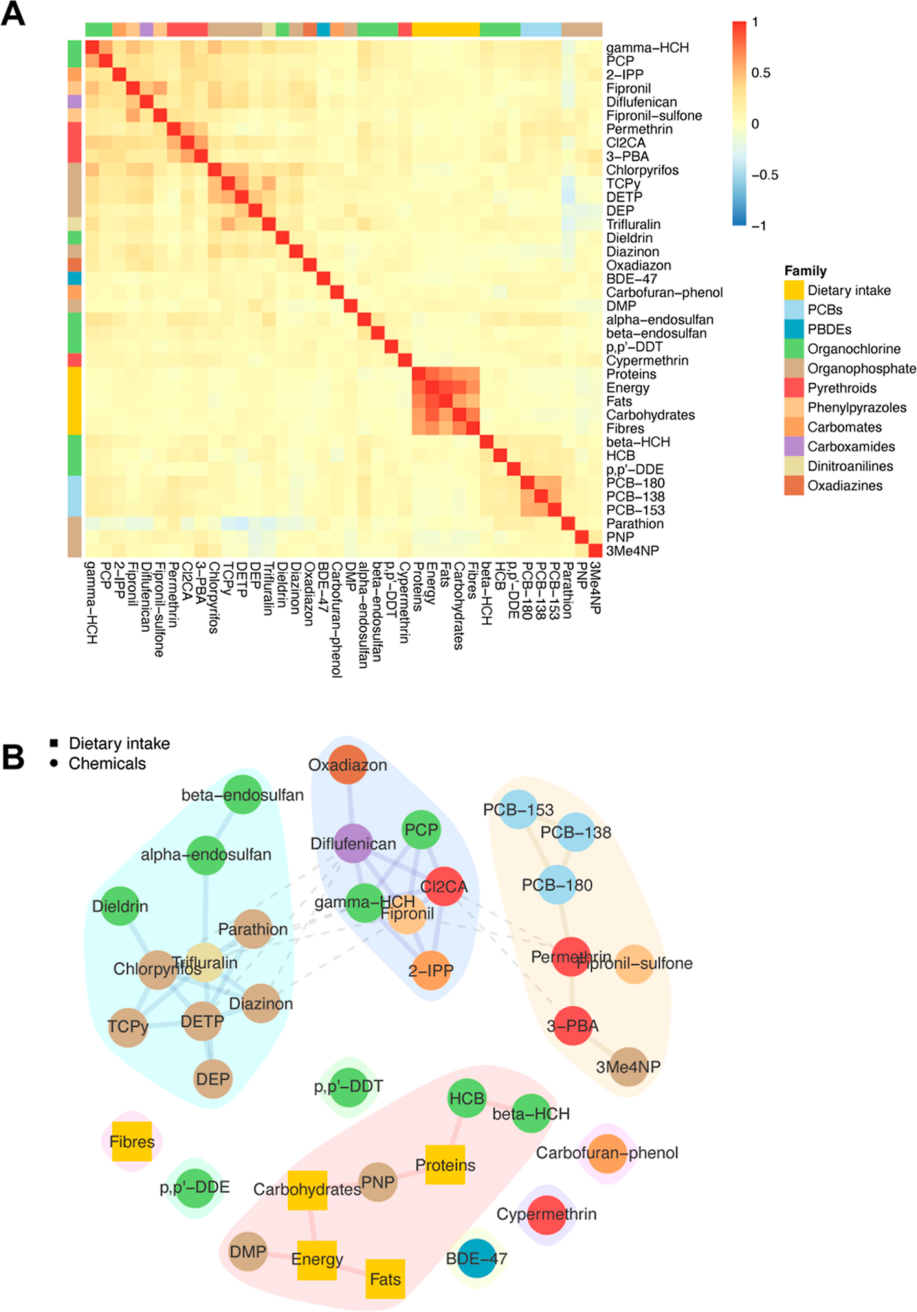
Descriptive summary of exposures in the 941 study participants. Heatmap representing pairwise Spearman’s correlation coefficient between dietary intake information and chemical measurements (A) and the corresponding conditional independence network and communities estimated using stability-enhanced graphical LASSO and the Louvain method, respectively (B).

Exposure information (dietary intake and chemical measurements) is summarized in Figure 2. A block-structure was observed in the pairwise correlation between the dietary intake variables, with the strongest correlation observed between energy and fat intake (ρ = 0.88). Moderate correlations were observed between chemicals of the same family or between the parent compound and its metabolites, such as 3-phenoxybenzoic acid (3-PBA) and *trans*-3-(2,2-dichlorovinyl)-2,2-dimethylcyclopropane carboxylic acid (Cl_2_CA) (ρ = 0.59), and fipronil and fipronil sulfone (ρ = 0.56) (Figure 2A). As previously reported, measurements derived from hair are not subject to the highly variable concentrations usually observed in fluids (blood and urine) and are therefore more accurate to capture chronic exposures, here spanning several months.^5,33^

To further investigate these correlation patterns, we estimated a conditional independence network using a multiblock (dietary intake and chemical measurements) stability-based calibration (Figure S3). The resulting network (Figure 2B) shows that the chemicals from the same family were often connected and belonged to the same community. Energy, fat, carbohydrate, and protein intake were directly or indirectly connected with dimethyl phosphate (DMP), *p*-nitrophenol (PNP), hexachlorobenzene (HCB), and beta-hexachlorocyclohexane (beta-HCH), with all chemicals grouped in the same community.

PCA was performed on the chemical measurements from hair, revealing complex and diverse exposure profiles across the population (Figure S4). The projection of exposure measurements indicated separation between participants from Belgium and Luxembourg, indicating potential environmental differences attributed to varying sources of pollutants. Linear regression models were used to examine the marginal association between each pollutant exposure and social or behavioral factors (Figure S5). In particular, we identified differential exposure to 13 and 9 pollutants in relation to age (Figure S5A) and gender (Figure S5B). We also found that individuals with low educational attainment had higher levels of HCB, parathion, and 3-PBA, and lower levels of chlorpyrifos, gamma-HCH, and diazinon, compared to those with high educational attainment (Figure S5C). Measured levels of Cl_2_CA and fipronil were found to be higher in smokers compared to nonsmokers (Figure S5D), and we identified differential levels of DMP, PNP, and HCB in relation to dietary patterns (Figure S5E).

These results support our adjustment in subsequent analyses for age, gender, educational attainment, smoking status, and diet, as summarized by the three first components of the PCA (jointly explaining >95% of the variance of the five dietary variables, see Table S5).

### Phenotype-Exposure Associations

Univariate analyses regressing the (*N* = 9) traits against the (*N* = 33) exposures separately (Table S6) showed association between levels of HCB in hair and BMI, WC, and HDL-C. Additionally, the levels of beta-HCH were associated with BMI and WC. Cypermethrin levels showed association with SBP and DBP, and three other exposures (parathion, fipronil, and diflufenican) were associated with DBP. Dieldrin levels were found to be associated with TC. Our results did not identify any exposures associated with FPG, TG, or LDL-C.

**Figure 3:**
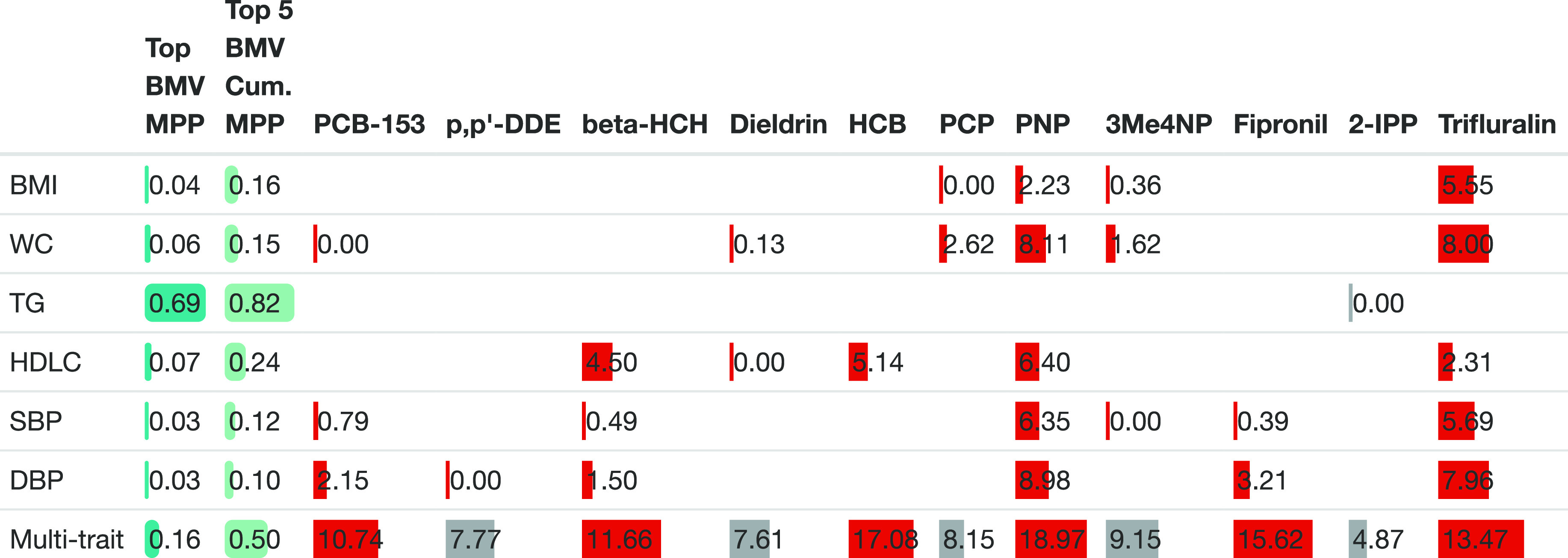
Comparison of results from the GUESS BVS approaches, adjusted for age, sex, educational attainment, smoking, and diet. The MPP of the top BMV and the top five BMV are indicated in the first two columns. Marginal strength of association of each exposure across single- and multitrait analyses is measured by the ratio of Bayes factors (RBF, reported here on the log_10_ scale for readability). Significant predictors based on an empirical FDR procedure are depicted in gray, whereas predictors that are also in the top BMV are indicated in red. Results for predictors with an MPPI lower than the threshold at FDR < 5% across the six single-trait analyses were omitted. *The BMV predicting TG was an empty model after adjusting for confounding factors.

The single-trait analyses using GUESS identified an outcome-specific sparse set of exposures jointly predicting each phenotype except for TG (Figure 3). Hair levels of PCP, PNP, 3Me4NP, and trifluralin were found to be jointly associated with BMI and WC. We found that dieldrin and, to a lesser extent, PCB-153 added additional information related to WC. SBP and DBP were jointly explained by PCB-153, beta HCH, PNP, fipronil, and trifluralin. Exposures with weaker associations (RBF ≤ 1) with SBP (3Me4NP and dieldrin) and with DBP (p,p′-DDE) were additionally selected in the top BMV for each outcome (Table S7). HDL-C was jointly explained by beta-HCH, HCB, PNP, trifluralin, and, to a lesser extent dieldrin. Overall, our BVS approach has been able to identify a sparse set of complementary exposures jointly explaining each phenotype, separately. While the combination of predictors selected were specific to each trait, we found that PNP and trifluralin were associated with most of the outcomes both showing the highest RBF across single-trait models (Table S7).

The six complementary traits (BMI, WC, TG, HDL-C, SBP, and DBP) that were central to the conditional independence network were set as outcomes for our main multitrait analysis. The model was run for 30,000 iterations, and the first 10,000 were discarded to account for burn-in. Trace plots showed good convergence of the algorithm and overlap of the different chains, indicating good exchange of information across chains (Figure S6). Based on RBF estimates (Table S7), we found that a total of 30 exposures contributed to the explanation of the multivariate outcome. By comparing the models visited in GUESS based on their posterior probability, a parsimonious set of six exposures (PNP, HCB, fipronil, trifluralin, beta-HCH, and PCB-153) were selected in the most supported model with an MPP of 0.16 (Figure 3).The RBF of these selected variables was higher than those of the same exposures in the single-trait analyses.

The multitrait analysis allowed detection of strong associations with combinations of preclinical or subclinical phenotypes that would have been missed by the single-trait analyses. For example, across the single-trait models, HCB was detected as a significant predictor of only HDL-C. However, the multitrait analysis revealed its strong association with the combination of the six traits, with a similar strength of association (RBF) to exposures (PNP and trifluralin) consistently associated with each trait. Although not in the BMV, several exposures (p,p′-DDE, 3Me4NP, and dieldrin) were more strongly associated with the multivariate phenotype than with any of the individual traits.

The MPP of the top BMV (as well as the cumulative MPP of the top five BMV) was higher in the multitrait analysis than in the single-trait analyses, suggesting that the multitrait model allows for a better explanation of the variance of the (albeit more complex) outcome. The correlation structures between chemical measurements, as observed in the exposure conditional independence network (Figure 2B), suggest that the best supported model excludes highly correlated predictors and only selects complementary exposures that best explain the multivariate outcome. Some exposures that were consistently detected in the single-trait analyses but missing from the multitrait analysis (e.g., dieldrin) are in close proximity with exposures included in the top BMV from the multitrait analysis (e.g., trifluralin).

Therefore, the multitrait, multiexposure associations provided by the top BMV in our approach enhance the interpretation of the results and the identification of exposures strongly associated with complex cardiometabolic outcomes. However, sparsity is not always equivalent to interpretability, and when prior information is available, data could be pruned to focus on features affecting a specific pathway of interest. Sparsity induced from our BVS approach on this selected set of (*N* = 33) pollutant measurements from hair has the potential to highlight the determinants driving the dysregulation of the pathway at a more granular level.

Considering all (*N* = 9), clinical phenotypes in our multitrait analysis resulted in a top BMV including four exposures (Table S7) and yielded a lower MPP 0.09. This indicates that the inclusion of less correlated traits to be jointly modeled as the multivariate outcome hampers the performance of the BVS approach in selecting a set of predictors, i.e., less supported models and weaker effects detected. Further sensitivity analyses (i) excluding TG from the multitrait outcome resulted in 3Me4NP additionally being selected in the top BMV, slightly increasing the MPP to 0.17, and (ii) including FPG in the outcome resulted in PCB-153 being excluded from the top BMV, which yielded an MPP of 0.18. While our approach appears as an efficient way to perform variable selection for complex (multivariate) outcomes, the effect of the correlation structure across outcomes is strongly affecting the performances of the model. As such, the prior selection of outcomes of interest should be carefully considered, and either prior knowledge^39^ or data exploration should drive this process. The identification of the optimal way to select the outcomes of interest should be further investigated by using simulated and real data sets. This would be key to establish our approach as a tool for exposome research in complex health outcomes.

The unadjusted model selected nine exposures as jointly predictive of cardiometabolic health. These included PCB-153, beta-HCH, gamma-HCH, HCB, DEP, permethrin, 3-PBA, diflufenican, and oxadiazon. Upon adjustment of age, sex, educational attainment, and smoking status, three exposures (PNP, fipronil, and trifluralin) were additionally selected, while six exposures (gamma-HCH, DEP, 3-PBA, difludenican, and oxadiazon) were no longer selected, suggesting that their effect could be explained by these adjustment factors. Out of the eight exposures selected before adjustment for dietary intake, six exposures (PCB-153, beta-HCH, HCB, PNP, fipronil, and trifluralin) were also selected in the fully adjusted model. The fact that 3Me4NP and permethrin were not selected after adjustment may indicate that these captured diet-related exposures. Although included in the fully adjusted model, the MPPI of PCB-153 dropped from 0.96 to 0.78 upon adjustment for dietary information. This suggests that some of its effect on the multitrait outcome could be explained by diet.

**Figure 4:**
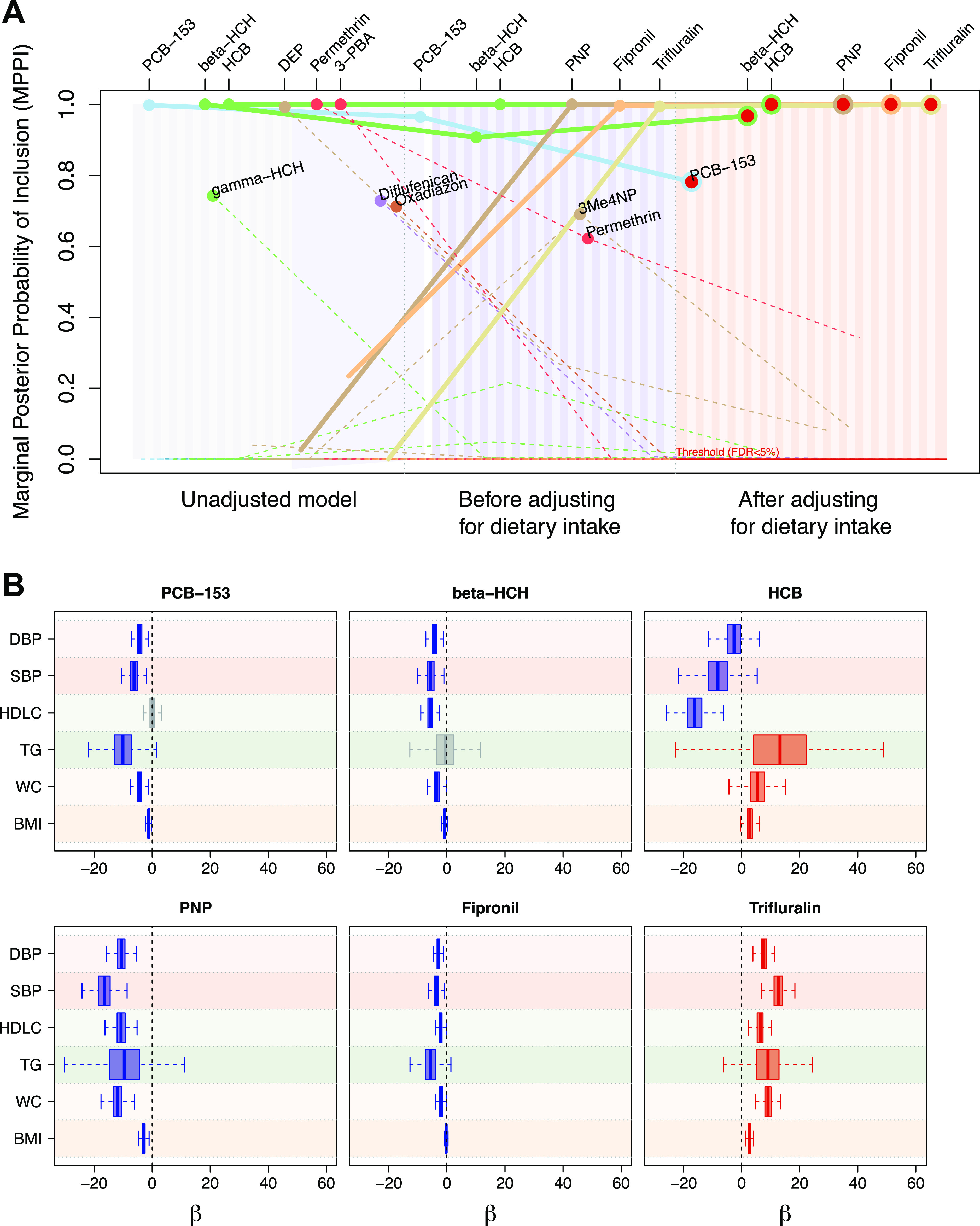
Comparison of the posterior strength of association in multitrait analysis as measured by the MPPI of each predictor in an unadjusted model and in models sequentially adjusted for (i) age and sex, educational attainment and smoking, and (ii) dietary information (A). The fully adjusted model corresponds to the main model reported. The predictors selected in the most supported multivariate model, top BMV, are indicated by a point. The lines represent the path of MPPI across the models for each predictor. The six predictors selected in the top BMV of the fully adjusted model are outlined in red with corresponding paths represented by solid lines; the path of all other predictors are dashed. Estimated posterior distribution of regression coefficients of predictors selected in the top BMV from the fully adjusted model is shown for each trait included in the multivariate outcome of the model (B). The direction of effect are indicated in red (positive) and blue (negative) for distributions, of which 25–75th percentiles do not include zero.

The effect size of each of the predictors selected in the top BMV was estimated from the fully adjusted model (Figure 4B). To estimate and quantify the predictive performance of the resulting model, data splitting (into training and testing sets) is warranted, and this could be implemented by refitting a regression model with the selected variables and estimated regression coefficients. Increased exposure to HCB and trifluralin was associated with an adverse cardiometabolic health profile, as evidenced by positive associations with obesity (higher BMI and WC) and dyslipidemia (higher TG). This is supported by previous evidence from a systematic review,^43^ which demonstrated positive associations between HCB and systemic arterial hypertension, peripheral arterial disease, and cardiovascular mortality, as well as between occupational exposure to trifluralin and risk of acute myocardial infarction. Experimental studies on rat models have also outlined an association between HCB exposure and CVD.^44^

The evidence concerning the association between increased exposures to HCB and trifluralin and HDL-C and blood pressure displayed varying degrees of clarity. Elevated exposure to HCB was associated with dyslipidemia, characterized by lower HDL-C levels, while trifluralin exposure appeared to have a protective effect (higher HDL-C levels). Moreover, increased exposures to trifluralin were associated with hypertension (higher SBP and DBP), whereas a reverse association was observed between HCB exposure and blood pressure. Our analysis also revealed inverse associations among exposure to PCB-153, PNP, fipronil, and beta-HCH and cardiometabolic health. Specifically, increased exposure to these compounds was linked with lower BMI, WC, and TG levels and blood pressure. Similarly, increased exposure to PNP, fipronil, and beta-HCH was associated with reduced levels of HDL-C. These inverse associations were also detected in our preliminary analyses relying on unviariate regression models and are to be interpreted very carefully. Our descriptive analyses highlighted that exposure to these compounds were associated with sociodemographic and dietary factors. Furthermore, our conditional independence networks indicated some correlation between exposures and dietary factors, and we observed some attenuation of the posterior strength of association between some exposures and cardiometabolic health outcomes after adjusting for the diet.

Altogether, these findings suggests that hair-derived measurements may capture potential residual confounding, wherein differential lifestyle behaviors influence exposure to compounds, consequently impacting health outcomes. For example, exposure to HCB, a highly fat-soluble pesticide which accumulates particularly in dairy products and animal meat,^45^ may be influenced by choices in (fatty) food intake. Thus, the association between HCB exposure and cardiometabolic health conditions identified in our study may be attributed to the effect of the pesticide HCB itself or unmeasured confounding by diet, both reflected in hair-derived measurements. Conversely, the inverse associations observed could be explained by increased pollutant exposure resulting from healthier lifestyle (e.g., healthy diet rich in fruit and vegetables), which may manifest as an overall improvement in cardiometabolic health. However, the formal assessment of these hypotheses was limited by data availability in the present study. To ensure external validity in other contexts (e.g., other countries), it is crucial to distinguish the effect of detailed dietary patterns from the effect of the environmental pollutants themselves. This warrants further investigations into the sources of exposure to pollutants measured from hair in more deeply characterized studies to disambiguate the associations between hair-derived measurements and cardiometabolic health outcomes. Addressing this distinction holds the potential utility of hair-derived measurements in complementing self-reported data when investigating specific external exposures, including diet, that contribute to cardiovascular risk profiles.

In conclusion, hair-derived measurements provide a valuable tool for characterizing embodiment of complex exposures and monitoring various risk factors of cardiometabolic health, at an individual level. We proposed a multitrait BVS approach to model the complexity of exposure profiles and cardiometabolic health by considering sets of complementary exposures and traits. By combining our BVS model with conditional independence networks, we identified a subset of six densely correlated traits (BMI, WC, TG, HDL-C, SBP, and DBP), and selected sets of exposures jointly predicting cardiometabolic health, as defined by these traits. Our approach was complemented by sets of single-trait analyses, which enabled the identification of exposures that were uniquely associated with specific traits as well as exposures that were consistently associated across all traits. The phenotype–exposure associations identified from the multitrait analysis exhibited overall stronger associations than those detected from single-trait analyses. Overall, this is suggestive of an increased statistical power yielded by the joint modeling of the correlated outcomes of interest. The use of multivariate outcome models, such as the one we proposed, enables us to capture the complexity of (aspects of) individual cardiometabolic health while providing sparse results with preserved interpretability.

## Supporting Information Available

Graphical flowchart of hierarchical connection of all data and models, summary of chemical compounds, user-defined parameter fields in GUESS approaches, overview of measured cardiometabolic traits, results from the PCA of dietary intake information, results from the single-trait univariate analyses, comparison of results from the GUESS Bayesian Variable Selection approaches, calibration plots of conditional independence networks, results from the PCA of hair-derived measurements, marginal covariate-exposure associations, and example of plots showing convergence of GUESS algorithm in multi-trait analysis PDF

## References

[ref1] Wild C. P. Complementing the Genome with an “Exposome”: The Outstanding Challenge of Environmental Exposure Measurement in Molecular Epidemiology. Cancer Epidemiol. Biomarkers Prev. 2005, 14, 1847–1850. 10.1158/1055-9965.EPI-05-0456. 16103423

[ref2] Wild C. P. The exposome: from concept to utility. Int. J. Epidemiol. 2012, 41, 24–32. 10.1093/ije/dyr236. 22296988

[ref3] Vermeulen R.; Schymanski E. L.; Barabási A. L.; Miller G. W. The exposome and health: Where chemistry meets biology. Science 2020, 367, 392–396. 10.1126/science.aay3164. 31974245 PMC7227413

[ref4] Appenzeller B. M.; Tsatsakis A. M. Hair analysis for biomonitoring of environmental and occupational exposure to organic pollutants: State of the art, critical review and future needs. Toxicol. Lett. 2012, 210, 119–140. 10.1016/j.toxlet.2011.10.021. 22079616

[ref5] Appenzeller B. M. R.; Hardy E. M.; Grova N.; Chata C.; Faÿs F.; Briand O.; Schroeder H.; Duca R.-C. Hair analysis for the biomonitoring of pesticide exposure: comparison with blood and urine in a rat model. Arch. Toxicol. 2017, 91, 2813–2825. 10.1007/s00204-016-1910-9. 28011991 PMC5515982

[ref6] Hardy E. M.; Duca R. C.; Salquebre G.; Appenzeller B. M. Multi-residue analysis of organic pollutants in hair and urine for matrices comparison. Forensic Sci. Int. 2015, 249, 6–19. 10.1016/j.forsciint.2014.12.003. 25553512

[ref7] Tsatsakis A.; Tzatzarakis M.; Tutudaki M.; Babatsikou F.; Alegakis A.; Koutis C. Assessment of levels of organochlorine pesticides and their metabolites in the hair of a Greek rural human population. Hum. Exp. Toxicol. 2008, 27, 933–940. 10.1177/0960327108102047. 19273549

[ref8] Peng F.-J.; Hardy E. M.; Béranger R.; Mezzache S.; Bourokba N.; Bastien P.; Li J.; Zaros C.; Chevrier C.; Palazzi P.; Soeur J.; Appenzeller B. M. Human exposure to PCBs, PBDEs and bisphenols revealed by hair analysis: A comparison between two adult female populations in China and France. Environ. Pollut. 2020, 267, 115425 10.1016/j.envpol.2020.115425. 32882460

[ref9] Peng F.-J.; Palazzi P.; Viguié C.; Appenzeller B. M. Measurement of hair thyroid and steroid hormone concentrations in the rat evidence endocrine disrupting potential of a low dose mixture of polycyclic aromatic hydrocarbons. Environ. Pollut. 2022, 313, 120179 10.1016/j.envpol.2022.120179. 36116566

[ref10] Ren M.; Yan L.; Pang Y.; Jia X.; Huang J.; Shen G.; Cheng H.; Wang X.; Pan B.; Li Z.; Wang B. External interference from ambient air pollution on using hair metal(loid)s for biomarker-based exposure assessment. Environ. Int. 2020, 137, 105584 10.1016/j.envint.2020.105584. 32106049

[ref11] Ren M.; Jia X.; Shi J.; Yan L.; Li Z.; Lan C.; Chen J.; Li N.; Li K.; Huang J.; Wu S.; Lu Q.; Li Z.; Wang B.; Liu J. Simultaneous analysis of typical halogenated endocrine disrupting chemicals and metal(loid)s in human hair. Sci. Total Environ. 2020, 718, 137300 10.1016/j.scitotenv.2020.137300. 32097838

[ref12] Ren M.; Fang M.; Liu J.; Lu Q.; Bao H.; Zhuang L.; Meng F.; Pan B.; Yan L.; Li Z.; et al. Applying hair exposome for linking environmental exposure to reproductive health: A comprehensive review and research perspective. Hyg. Environ. Health Adv. 2024, 9, 100086 10.1016/j.heha.2023.100086.

[ref13] Visseren F. L. J.; Mach F.; Smulders Y. M.; Carballo D.; Koskinas K. C.; Bäck M.; Benetos A.; Biffi A.; Boavida J. M.; Capodanno D.; et al. 2021 ESC Guidelines on cardiovascular disease prevention in clinical practice: Developed by the Task Force for cardiovascular disease prevention in clinical practice with representatives of the European Society of Cardiology and 12 medical societies With the special contribution of the European Association of Preventive Cardiology (EAPC). Eur. Heart J. 2021, 42, 3227–3337. 10.1093/eurheartj/ehab484. 34458905

[ref14] Cosselman K. E.; Navas-Acien A.; Kaufman J. D. Environmental factors in cardiovascular disease. Nat. Rev. Cardiol. 2015, 12, 627–642. 10.1038/nrcardio.2015.152. 26461967

[ref15] Bhatnagar A. Cardiovascular pathophysiology of environmental pollutants. Am. J. Physiol.-Heart Circ. Physiol. 2004, 286, H479–H485. 10.1152/ajpheart.00817.2003. 14715496

[ref16] Al-Kindi S. G.; Brook R. D.; Biswal S.; Rajagopalan S. Environmental determinants of cardiovascular disease: lessons learned from air pollution. Nat. Rev. Cardiol. 2020, 17, 656–672. 10.1038/s41569-020-0371-2. 32382149 PMC7492399

[ref17] Solenkova N. V.; Newman J. D.; Berger J. S.; Thurston G.; Hochman J. S.; Lamas G. A. Metal pollutants and cardiovascular disease: Mechanisms and consequences of exposure. Am. Heart J. 2014, 168, 812–822. 10.1016/j.ahj.2014.07.007. 25458643 PMC4254412

[ref18] Lind L.; Lind P. M. Can persistent organic pollutants and plastic-associated chemicals cause cardiovascular disease?. J. Intern. Med. 2012, 271, 537–553. 10.1111/j.1365-2796.2012.02536.x. 22372998

[ref19] Perkins J. T.; Petriello M. C.; Newsome B. J.; Hennig B. Polychlorinated biphenyls and links to cardiovascular disease. Environ. Sci. Pollut. Res. 2016, 23, 2160–2172. 10.1007/s11356-015-4479-6. PMC4609220 25877901

[ref20] Evangelou E.; Ntritsos G.; Chondrogiorgi M.; Kavvoura F. K.; Hernández A. F.; Ntzani E. E.; Tzoulaki I. Exposure to pesticides and diabetes: A systematic review and meta-analysis. Environ. Int. 2016, 91, 60–68. 10.1016/j.envint.2016.02.013. 26909814

[ref21] Czajka M.; Matysiak-Kucharek M.; Jodłowska-Jędrych B.; Sawicki K.; Fal B.; Drop B.; Kruszewski M.; Kapka-Skrzypczak L. Organophosphorus pesticides can influence the development of obesity and type 2 diabetes with concomitant metabolic changes. Environ. Res. 2019, 178, 108685 10.1016/j.envres.2019.108685. 31479978

[ref22] Hernández-Mariano J. Á.; Baltazar-Reyes M. C.; Salazar-Martínez E.; Cupul-Uicab L. A. Exposure to the pesticide DDT and risk of diabetes and hypertension: Systematic review and meta-analysis of prospective studies. Int. J. Hyg. Environ. Health 2022, 239, 113865 10.1016/j.ijheh.2021.113865. 34700204

[ref23] Alkerwi A.; Guillaume M.; Zannad F.; Laufs U.; Lair M.-L. Nutrition, environment and cardiovascular health (NESCAV): protocol of an inter-regional cross-sectional study. BMC Public Health 2010, 10, 698 10.1186/1471-2458-10-698. 21078172 PMC2998492

[ref24] Grundy S. M.; Brewer H. B.; Cleeman J. I.; Smith S. C.; Lenfant C.; Franklin B. A.; Gordon D. J.; Krauss R. M.; Savage P. J.; Smith Jr S. C.; et al. Definition of metabolic syndrome: Report of the National Heart, Lung, and Blood Institute/American Heart Association conference on scientific issues related to definition. Circulation 2004, 109, 433–438. 10.1161/01.cir.0000111245.75752.c6. 14744958

[ref25] Grundy S. M.; Cleeman J. I.; Daniels S. R.; Donato K. A.; Eckel R. H.; Franklin B. A.; Gordon D. J.; Krauss R. M.; Savage P. J.; Smith S. C.; Spertus J. A.; Costa F. Diagnosis and Management of the Metabolic Syndrome. Circulation 2005, 112, 2735–2752. 10.1161/CIRCULATIONAHA.105.169404. 16157765

[ref26] Alkerwi A.; Sauvageot N.; Donneau A.-F.; Lair M.-L.; Couffignal S.; Beissel J.; Delagardelle C.; Wagener Y.; Albert A.; Guillaume M. First nationwide survey on cardiovascular risk factors in Grand-Duchy of Luxembourg (ORISCAV-LUX). BMC Public Health 2010, 10, 468 10.1186/1471-2458-10-468. 20698957 PMC2925827

[ref27] Alkerwi A.; Vernier C.; Sauvageot N.; Crichton G. E.; Elias M. F. Demographic and socioeconomic disparity in nutrition: application of a novel Correlated Component Regression approach. BMJ Open 2015, 5, e006814 10.1136/bmjopen-2014-006814. PMC4431064 25967988

[ref28] Sauvageot N.; Alkerwi A.; Albert A.; Guillaume M. Use of food frequency questionnaire to assess relationships between dietary habits and cardiovascular risk factors in NESCAV study: validation with biomarkers. Nutr. J. 2013, 12, 143 10.1186/1475-2891-12-143. 24195492 PMC4176104

[ref29] Streel S.; Donneau A. F.; Hoge A.; Majerus S.; Kolh P.; Chapelle J. P.; Albert A.; Guillaume M.; Guillaume M. Socioeconomic Impact on the Prevalence of Cardiovascular Risk Factors in Wallonia, Belgium: A Population-Based Study. BioMed Res. Int. 2015, 2015, 1–10. 10.1155/2015/580849. PMC4561934 26380280

[ref30] Peng F.-J.; Emond C.; Hardy E. M.; Sauvageot N.; Alkerwi A.; Lair M.-L.; Appenzeller B. M. Population-based biomonitoring of exposure to persistent and non-persistent organic pollutants in the Grand Duchy of Luxembourg: Results from hair analysis. Environ. Int. 2021, 153, 106526 10.1016/j.envint.2021.106526. 33839549

[ref31] Peng F.-J.; Lin C.-A.; Wada R.; Bodinier B.; Iglesias-González A.; Palazzi P.; Streel S.; Guillaume M.; Vuckovic D.; Chadeau-Hyam M.; Appenzeller B. M. Association of hair polychlorinated biphenyls and multiclass pesticides with obesity, diabetes, hypertension and dyslipidemia in NESCAV study. J. Hazard. Mater. 2024, 461, 132637 10.1016/j.jhazmat.2023.132637. 37788552

[ref32] Kintz P.; Salomone A.; Vincenti M. Hair Analysis in Clinical and Forensic Toxicology; Academic Press, 2015.

[ref33] Hardy E. M.; Dereumeaux C.; Guldner L.; Briand O.; Vandentorren S.; Oleko A.; Zaros C.; Appenzeller B. M. Hair versus urine for the biomonitoring of pesticide exposure: Results from a pilot cohort study on pregnant women. Environ. Int. 2021, 152, 106481 10.1016/j.envint.2021.106481. 33706039

[ref34] Stekhoven D. J.; Bühlmann P. MissForest—non-parametric missing value imputation for mixed-type data. Bioinformatics 2012, 28, 112–118. 10.1093/bioinformatics/btr597. 22039212

[ref35] Bodinier B. sharp: Stability-enHanced Approaches using Resampling Procedures. R package version 1.3.0, 2022.

[ref36] Bodinier B.; Filippi S.; Nøst T. H.; Chiquet J.; Chadeau-Hyam M. Automated calibration for stability selection in penalised regression and graphical models. J. R. Stat. Soc. C 2023, 72, qlad058. 10.1093/jrsssc/qlad058 PMC10746547 38143734

[ref37] Blondel V. D.; Guillaume J.-L.; Lambiotte R.; Lefebvre E. Fast unfolding of communities in large networks. J. Stat. Mech.: Theory Exp. 2008, 2008, P10008 10.1088/1742-5468/2008/10/P10008.

[ref38] Bottolo L.; Chadeau-Hyam M.; Hastie D. I.; Langley S. R.; Petretto E.; Tiret L.; Tregouet D.; Richardson S. ESS++: a C++ objected-oriented algorithm for Bayesian stochastic search model exploration. Bioinformatics 2011, 27, 587–588. 10.1093/bioinformatics/btq684. 21233165 PMC3035799

[ref39] Bottolo L.; Chadeau-Hyam M.; Hastie D. I.; Zeller T.; Liquet B.; Newcombe P.; Yengo L.; Wild P. S.; Schillert A.; Ziegler A.; et al. GUESS-ing Polygenic Associations with Multiple Phenotypes Using a GPU-Based Evolutionary Stochastic Search Algorithm. PLOS Genet. 2013, 9, e1003657 10.1371/journal.pgen.1003657. 23950726 PMC3738451

[ref40] Liquet B.; Bottolo L.; Campanella G.; Richardson S.; Chadeau-Hyam M. R2GUESS: A Graphics Processing Unit-Based R Package for Bayesian Variable Selection Regression of Multivariate Responses. J. Stat. Software 2016, 69, 1–32. 10.18637/jss.v069.i02. PMC5860753 29568242

[ref41] Chipman H.; George E. I.; McCulloch R. E. Institute of Mathematical Statistics Lecture Notes – Monograph Series; Lecture Notes-Monograph Series; Institute of Mathematical Statistics: Beachwood, OH, 2001, pp 65–116.

[ref42] Kass R. E.; Raftery A. E. Bayes Factors. J. Am. Stat. Assoc. 1995, 90, 773–795. 10.1080/01621459.1995.10476572.

[ref43] Zago A. M.; Faria N. M. X.; Fávero J. L.; Meucci R. D.; Woskie S.; Fassa A. G. Pesticide exposure and risk of cardiovascular disease: A systematic review. Glob. Public Health 2022, 17, 3944–3966. 10.1080/17441692.2020.1808693. 32816635

[ref44] Castilla R.; Asuaje A.; Rivière S.; Romero C. G.; Martín P.; Cao G.; Kleiman de Pisarev D.; Milesi V.; Alvarez L. Environmental pollutant hexachlorobenzene induces hypertension in a rat model. Chemosphere 2018, 195, 576–584. 10.1016/j.chemosphere.2017.11.117. 29277037

[ref45] Barber J. L.; Sweetman A. J.; van Wijk D.; Jones K. C. Hexachlorobenzene in the global environment: Emissions, levels, distribution, trends and processes. Sci. Total Environ. 2005, 349, 1–44. 10.1016/j.scitotenv.2005.03.014. 16005495

