# Supplementary material for: Hair-Derived Exposome Exploration of Cardiometabolic Health: Piloting a Bayesian Multitrait Variable Selection Approach
Source: Environ Sci Technol. 2024 Mar 13;58(12):5383–93. doi: 10.1021/acs.est.3c08739 (PMC10976885; doi:10.1021/acs.est.3c08739)
Supplement: Supplementary file 1 — es3c08739_si_001.pdf [file es3c08739_si_001.pdf]

Supporting Information: Hair-derived exposome exploration of cardiometabolic health: piloting a Bayesian multi-trait variable selection approach

Rin Wada,[†,‡] Feng-Jiao Peng,[¶] Chia-An Lin,[†] Roel Vermeulen,[†,§] Alba Iglesias-González,[¶] Paul Palazzi,[¶] Barbara Bodinier,[†,‡] Sylvie Streel,[‖] Michèle Guillaume,[‖] Dragana Vuckovic,[†,‡] Sonia Dagnino,[†,⊥] Julien Chiquet,[#] Brice M. R. Appenzeller,[¶] and Marc Chadeau-Hyam[*,†,‡]

†*Department of Epidemiology and Biostatistics, School of Public Health, Imperial College London, London, W2 1PG, UK*

‡*MRC Centre for Environment and Health, Imperial College London, London, W2 1PG, UK*

¶*Human Biomonitoring Research Unit, Department of Precision Health, Luxembourg Institute of Health, Strassen, L-1445, Luxembourg*

§*Institute for Risk Assessment Sciences, Utrecht University, Utrecht, 3584 CM, The Netherlands*

‖*Department of Public Health Sciences, University of Liege, Liege, 4000, Belgium*

⊥*Transporters in Imaging and Radiotherapy in Oncology (TIRO), Institut des sciences du vivant Fréderic Joliot, CEA, Université Côte d'Azur, Nice, 06107, France*

#*Université Paris-Saclay, AgroParisTech, INRAE, UMR MIA Paris-Saclay, Palaiseau, 91120, France*

E-mail: m.chadeau@imperial.ac.uk

Number of pages: 12

Number of figures: 6

Number of tables: 7

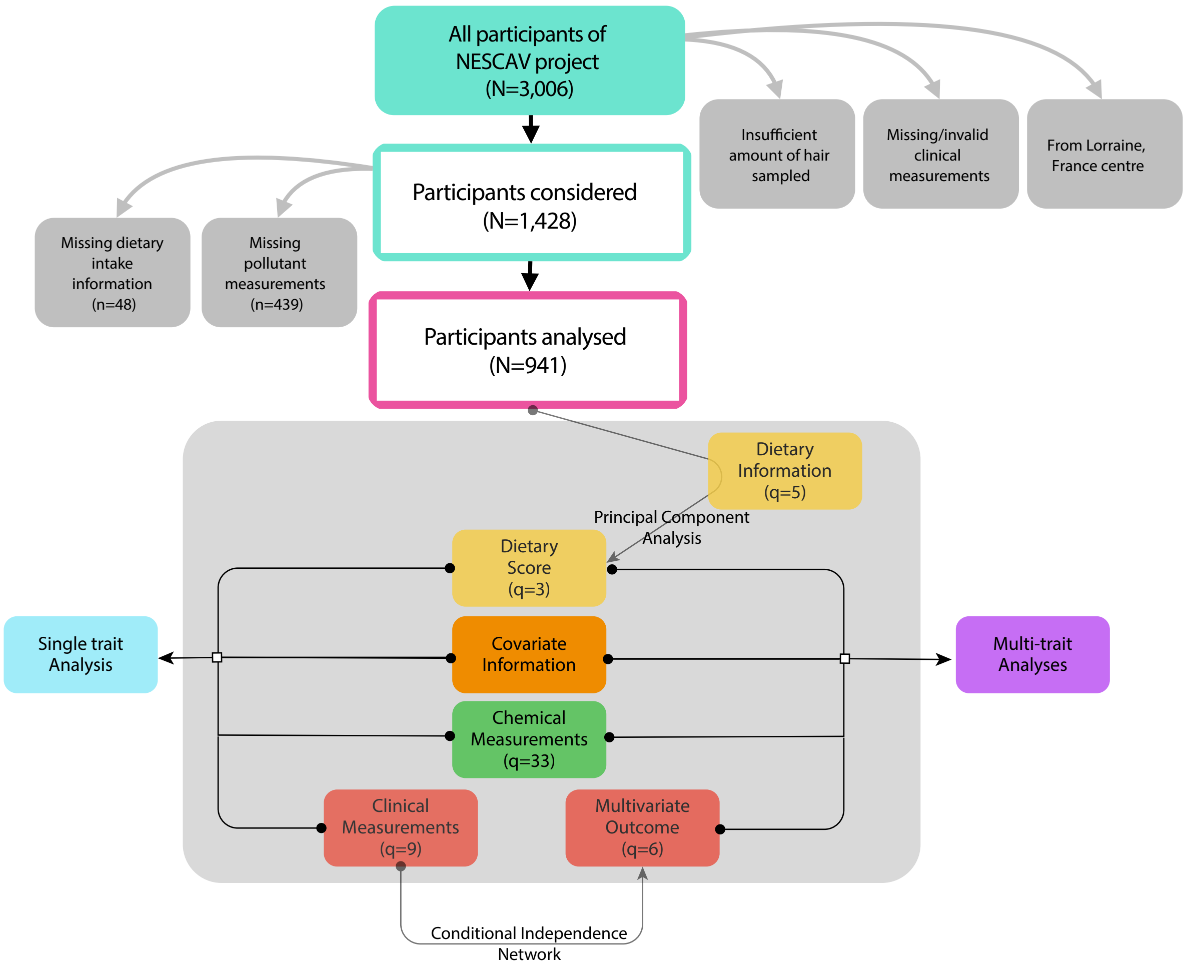

Figure S1: A graphical flowchart indicating how all data and models are hierarchically connected.

Table S1: A table containing the full names, abbreviations, and CAS numbers of all compounds. Metabolites are indicated with the parent compound(s).

Terminology	Abbreviation	CAS	Metabolite
Polychlorinated biphenyls (PCBs)			
Polychlorinated biphenyl 138	PCB-138	35065-28-2	
Polychlorinated biphenyl 153	PCB-153	35065-27-1	
Polychlorinated biphenyl 180	PCB-180	35065-29-3	
Polybrominated diphenyl ethers (PBDEs)			
brominated diphenyl ether 47	BDE-47	5436-43-1	
Organochlorine pesticides (OCs)			
p,p'-dichlorodiphenyltrichloroethane	p,p'-DDT	50-29-3	
p,p'-dichlorodiphenyldichloroethylene	p,p'-DDE	72-55-9	Metabolite (p,p'-DDT)
alpha-endosulfan		959-98-8	
beta-endosulfan		33213-65-9	
beta-hexachlorocyclohexane	beta-HCH	319-85-7	
gamma-hexachlorocyclohexane	gamma-HCH	58-89-9	
Dieldrin		60-57-1	
hexachlorobenzene	HCB	118-74-1	
pentachlorophenol	PCP	87-86-5	Metabolite (Chlorophenol)
Organophosphorus pesticides (OPs)			
Diazinon		333-41-5	
Chlorpyrifos		2921-88-2	
Parathion		56-38-2	
3, 5, 6-trichloro-2-pyridinol	TCPy	6515-38-4	Metabolite (Chlorpyrifos)
dimethyl phosphate	DMP	813-78-5	Metabolite (non-specific OP)
diethylphosphate	DEP	598-02-7	Metabolite (non-specific OP)
diethylthiophosphate	DETP	5871-17-0	Metabolite (non-specific OP)
para-nitrophenol	PNP	100-02-7	Metabolite (Parathion)
3-methyl-4-nitrophenol	3Me4NP	2581-34-2	Metabolite (Fenitrothion)
Pyrethroid pesticides (PYs)			
Permethrin		52645-53-1	
Cypermethrin		52315-07-8	
trans-3-(2,2-dichlorovinyl)-2,2-dimethylcyclopropane-carboxylic acid	Cl2CA	55701-05-8	Metabolite (non-specific PY)
3-phenoxybenzoic acid	3-PBA	3739-38-6	Metabolite (non-specific PY)
Phenylpyrazole pesticides (PHs)			
Fipronil		120068-37-3	
Fipronil sulfone		120068-36-2	Metabolite (Fipronil)
Carbamate pesticides (CAs)			
2-isopropoxyphenol	2-IPP	4812-20-8	Metabolite (Propoxur)
Carbofuran phenol		16655-82-6	Metabolite (Carbofuran)
Carboxamides			
Diflufenican		83164-33-4	
Dinitroanilines			
Trifluralin		1582-09-8	
Oxadiazines			
Oxadiazon		19666-30-9	

Table S2: Summary of chemical compounds included in study. Value of limit of detection (LOD) and range of detection (minimum, first quartile, median, third quartile, and maximum) are reported in concentrations (pg/mg). Proportion of detected values and missing values are reported for each chemical measurement.

	LOD	detected	Min	Q1	Q2	Q3	Max	missing
PCBs								
PCB-138	1.00	0.30	1.44e-03	0.32	0.65	1.20	27.00	0.00
PCB-153	1.03	0.32	1.47e-03	0.33	0.67	1.29	30.48	0.00
PCB-180	1.00	0.35	1.44e-03	0.34	0.71	1.70	40.31	0.00
PBDEs								
BDE-47	0.10	0.14	1.64e-04	0.03	0.06	0.09	262.77	0.00
Organochlorine								
p,p'-DDT	1.03	0.11	1.47e-03	0.27	0.52	0.81	168.55	0.00
p,p'-DDE	2.03	0.16	2.02e-03	0.39	0.81	1.46	4081.97	0.06
alpha-endosulfan	1.01e-02	0.82	1.80e-05	0.02	0.05	0.11	11.69	0.00
beta-endosulfan	0.20	0.14	3.36e-04	0.06	0.12	0.17	108.46	0.00
beta-HCH	0.50	0.26	8.09e-04	0.17	0.33	0.51	200.38	0.00
gamma-HCH	0.02	1.00	0.02	0.32	0.59	1.19	98.82	0.00
Dieldrin	0.20	0.13	3.37e-04	0.06	0.12	0.17	26.70	0.00
HCB	0.04	1.00	0.04	0.11	0.14	0.18	0.86	0.00
PCP	0.14	0.97	0.02	2.73	7.71	23.47	1681.65	0.00
Organophosphate								
Diazinon	0.21	0.21	3.50e-04	0.07	0.13	0.20	175.26	0.00
Chlorpyrifos	1.03e-02	0.89	1.67e-03	0.14	0.30	0.63	159.42	0.10
Parathion	1.00e-02	0.69	1.69e-05	7.40e-03	0.04	0.19	5.47	0.00
TCPy	0.50	0.85	2.50e-03	1.55	3.91	10.16	1910.35	0.10
DMP	1.00	0.21	1.44e-03	0.29	0.59	0.95	858.89	0.03
DEP	0.10	0.88	1.34e-02	0.50	1.44	4.53	347.13	0.10
DETP	1.02	0.35	1.46e-03	0.36	0.75	1.82	142.33	0.10
PNP	4.46	1.00	4.46	10.36	14.34	22.44	3396.44	0.00
3Me4NP	0.11	0.83	1.93e-04	0.31	0.82	1.83	103.29	0.00
Pyrethroids								
Permethrin	0.38	0.76	6.24e-04	1.18	13.33	48.76	38638.69	0.00
Cypermethrin	0.20	0.13	3.35e-04	0.06	0.12	0.17	40.49	0.00
Cl2CA	0.10	0.97	4.17e-03	0.67	1.67	4.61	320.38	0.00
3-PBA	1.05e-02	0.91	5.44e-05	0.20	0.41	0.95	105.27	0.01
Phenylpyrazoles								
Fipronil	0.20	0.74	3.36e-04	0.20	0.93	3.12	11649.66	0.01
Fipronil-sulfone	0.02	0.94	9.52e-05	0.23	0.65	2.47	223.39	0.00
Carbomates								
2-IPP	0.06	0.68	1.09e-04	0.05	1.46	7.09	2584.42	0.00
Carbofuran-phenol	0.50	0.15	8.14e-04	0.15	0.29	0.44	493.55	0.00
Carboxamides								
Diflufenican	0.10	0.55	1.69e-04	0.05	0.12	0.31	130.21	0.00
Dinitroanilines								
Trifluralin	1.18e-03	1.00	1.18e-03	0.05	0.10	0.17	20.55	0.00
Oxadiazines								
Oxadiazon	0.10	0.25	1.70e-04	0.03	0.07	0.10	111.40	0.00

Table S3: User-defined parameter fields in GUESS approaches.

Parameter	Value	Description
General parameters		
MAX_P_GAM_FACTOR	7	Factor (F) defining maximal model size (p_{max}).
Parameters of stepwise regression		
N_P_VALUE_ENTER	0.05	Maximum nominal p value for a term to be added.
N_P_VALUE_REMOVE	0.05	Minimum nominal p value for a term to be removed.
Setup parameters for moves		
GIBBS_N_BATCH	500	Number of sweeps between two full Gibbs scans.
P_MUTATION	0.5	Probability to perform FSMH move at each sweep.
P_SEL	0.5	Threshold on cumulative Boltzmann weights.
P_CSRV_R	0.375	Threshold for correlation coefficient ρ_0 to be considered in block crossover move.
K_MAX	2	Maximum number of breakpoints in the crossover move.
P_DR	0.5	Probability to perform a DR move among two possible exchange moves.
G_ADMH_OPTIMAL	0.44	Target acceptance rate for τ.
G_N_BATCH	100	Number of sweeps between two adaptations of standard deviation of proposal for τ.
G_ADMH_LS	0	Initial value for log standard deviation of τ proposal.
G_M_MIN	-1.22	Lower bound for log standard deviation of τ proposal. $-log(p/22)$, where $p = 33$.
G_M_MAX	1.22	Upper bound for log standard deviation of τ proposal. $log(p/22)$, where $p = 33$.
B_T	2	Initial value for argument b for temperature ladder.
A_T_DEN_INF_5K	2	Initial value for argument a for temperature ladder.
TEMP_N_BATCH	50	Number of DR moves between temperature placement.
TEMP_OPTIMAL	0.5	Optimal acceptance rate for DR move.
M_MIN	1	Lower bound for value of b_t in temperature placement.
M_MAX	4	Upper bound for value of b_t in temperature placement.

Table S4: Overview of measured cardiometabolic traits stratified by assessment centre. The proportion of missing values are shown for variables with missing values. SD = standard deviation. HDL-C = high-density lipoprotein cholesterol. LDL-C = low lipoprotein cholesterol.

	Total N=941	Belgium N=481	Luxembourg N=460
Body Mass Index, N (%)			
Underweight	19 (2.0%)	13 (2.7%)	6 (1.3%)
Normal	452 (48.0%)	250 (52.0%)	202 (43.9%)
Pre-obesity	282 (30.0%)	132 (27.4%)	150 (32.6%)
Obesity class I	129 (13.7%)	56 (11.6%)	73 (15.9%)
Obesity class II	47 (5.0%)	26 (5.4%)	21 (4.6%)
Obesity class III	12 (1.3%)	4 (0.8%)	8 (1.7%)
Waist circumference, N (%)			
Normal	666 (70.8%)	368 (76.5%)	298 (64.8%)
High	275 (29.2%)	113 (23.5%)	162 (35.2%)
Triglyceride, N (%)			
Normal	798 (84.8%)	423 (87.9%)	375 (81.5%)
High	143 (15.2%)	58 (12.1%)	85 (18.5%)
Total cholesterol (mg/dL), Mean (SD)	199.1 (39.5)	199.2 (39.2)	199.1 (39.7)
HDL-C (mg/dL), Mean (SD)	63.8 (17.2)	65.0 (17.2)	62.6 (17.2)
LDL-C (mg/dL), Mean (SD)	120.2 (34.6)	118.5 (34.6)	122.0 (34.6)
Systolic blood pressure (mmHg), Mean (SD)	126.8 (17.3)	124.0 (16.6)	129.7 (17.6)
Diastolic blood pressure (mmHg), Mean (SD)	79.3 (11.4)	76.3 (10.8)	82.4 (11.2)
Fasting plasma glucose (mg/dL), Mean (SD)	92.4 (20.5)	89.7 (21.2)	95.2 (19.3)

Table S5: Dietary intake information was summarised using the scores of the first three Principal Components (PC_1, PC_2, and PC_3) based on five numeric variables: energy (kcal/day), proteins (g/day), fats (g/day), carbohydrates (g/day), and fibres (g/day). The explained variance and loading of each variable are presented for each component.

	PC_1	PC_2	PC_3	PC_4	PC_5
**Explained variance**	75.5%	12.8%	6.6%	4.7%	0.4%
Energy	0.50	-0.15	-0.23	-0.08	-0.82
Proteins	0.44	-0.31	0.71	-0.42	0.16
Fats	0.44	-0.52	-0.24	0.58	0.38
Carbohydrates	0.45	0.36	-0.51	-0.49	0.41
Fibres	0.4	0.7	0.35	0.48	-0.03

Table S6: Results from the single-trait univariate analyses. A series of linear regression (33×9 models) was run to model the association between each measured cardiometabolic trait and each hair-derived measurement of pollutant exposure. Models were all adjusted for age, sex, smoking status and educational attainment. For clarity we report, for each outcome separately, the effect size estimates (and corresponding p-value) only for exposures found associated with that outcome, at a Bonferroni-corrected significance level ensuring a family wise error rate below 0.05.

Outcome	Significant predictor	p-value	β (95% CI)
BMI	beta-HCH	6.02e-06	0.70 (0.40,1.00)
	HCB	6.24e-13	1.11 (0.81,1.41)
WC	beta-HCH	5.06e-06	1.76 (1.01,2.51)
	HCB	1.68e-13	2.85 (2.11,3.60)
TC	Dieldrin	1.41e-03	-3.84 (-6.19,-1.49)
HDL-C	HCB	5.13e-05	-2.18 (-3.23,-1.13)
SBP	Cypermethrin	3.81e-04	-1.69 (-2.61,-0.76)
DBP	Parathion	1.28e-03	1.10 (0.43,1.76)
	Cypermethrin	9.10e-04	-1.12 (-1.77,-0.46)
	Fipronil	1.32e-03	-1.10 (-1.76,-0.43)
	Diflufenican	2.16e-04	-1.25 (-1.91,-0.59)

Table S7: Comparison of results from the GUESS Bayesian Variable Selection approaches. The Model Posterior Probability (MPP) of the top Best Models Visited (BMV) and the top five BMV are indicated in the first two rows, respectively. Comparison of the marginal strength of association across single-trait and multi-trait analyses is possible by a new re-scaled measure, Ratio of Bayes Factors (RBF). Significant predictors based on an empirical FDR procedure have RBF$\geq$ 1, whereas predictors that are also in the top BMV are indicated by the asterisks.

Outcome(s)	BMI	WC	TG	TC	LDLC	HDLC	SBP	DBP	FPG	Multi-trait	All traits	Excluding TG	Including FPG
Top BMV MPP	0.04	0.06	0.69	0.03	0.07	0.07	0.03	0.03	0.06	0.16	0.09	0.17	0.18
Top 5 BMV Cum. MPP	0.16	0.15	0.82	0.12	0.23	0.24	0.12	0.1	0.17	0.5	0.37	0.52	0.63
PCB-138	< 1	< 1	< 1	< 1	< 1	< 1	< 1	< 1	< 1	2.9e+06	2.7e+03	4.1e+05	4.2e+05
PCB-153	< 1	1*	< 1	< 1	< 1	< 1	6.1*	1.4e+02*	< 1	5.5e+10*	3.8e+07	2.1e+10*	1.2e+10
PCB-180	< 1	< 1	< 1	< 1*	1*	< 1	< 1	< 1	< 1	2.3e+04	2.1e+03	4.9e+03	5e+03
BDE-47	< 1	< 1	< 1	< 1	< 1	< 1	< 1	< 1	< 1	25	< 1	18	< 1
p,p'-DDT	< 1	< 1	< 1	< 1	< 1	< 1	< 1	< 1	< 1	3.8	< 1	16	1
p,p'-DDE	< 1	< 1	< 1	34*	< 1	< 1	< 1	1*	< 1	5.9e+07	5.9e+09	1e+06	1.4e+08
alpha-endosulfan	< 1	< 1	< 1	< 1	< 1	< 1	< 1	< 1	< 1	1.7e+02	2.2e+02	6.4e+02	< 1
beta-endosulfan	< 1	< 1	< 1	< 1	< 1	< 1	< 1	< 1	< 1	1	< 1	13	< 1
beta-HCH	< 1	< 1	< 1	1.1e+04*	13*	3.1e+04*	3.1*	31*	< 1	4.6e+11*	3.5e+09	5.2e+11*	1.8e+10*
gamma-HCH	< 1	< 1	< 1	< 1	< 1	< 1	< 1	< 1	< 1	5.5e+05	3.8e+06	2.2e+05	7.3e+05
Dieldrin	< 1	1.4*	< 1	2.1e+03*	49*	1*	< 1*	< 1	1.4*	4.1e+07	8.8e+06	6e+06	1.7e+07
HCB	< 1	< 1	< 1	< 1*	< 1	1.4e+05*	< 1	< 1	< 1	1.2e+17*	2.4e+14*	8.2e+24*	1.1e+16*
PCP	1*	4.2e+02*	< 1	< 1	< 1	< 1	< 1	< 1	< 1	1.4e+08	4.7e+07	3.4e+07	1.1e+07
Diazinon	< 1	< 1	< 1	< 1	< 1	< 1	< 1	< 1	< 1	< 1	3.5	24	6.1
Chlorpyrifos	< 1	< 1	< 1	< 1	< 1	< 1	< 1	< 1	< 1	2.8e+03	< 1	3.5e+03	1.3e+02
Parathion	< 1	< 1	< 1	< 1	< 1	< 1	< 1	< 1	< 1	2.8e+04	5.9e+08	7.9e+04	9.2e+03
TCPy	< 1	< 1	< 1	< 1	< 1	< 1	< 1	< 1	< 1	3.9e+02	< 1	68	< 1
DMP	< 1	< 1	< 1	< 1	< 1	< 1	< 1	< 1	< 1	< 1	< 1	1	< 1
DEP	< 1	< 1	< 1	< 1	< 1	< 1	< 1*	< 1	< 1	1.4e+09	7.3e+09*	4.4e+08	1.3e+09
DETP	< 1	< 1	< 1	< 1	< 1	< 1	< 1	< 1	< 1	< 1	1	37	< 1
PNP	1.7e+02*	1.3e+08*	< 1	4.8e+11*	1.7e+03*	2.5e+06*	2.2e+06*	9.6e+08*	2.3e+02*	9.3e+18*	2e+19*	8e+15*	6.1e+18*
3Me4NP	2.3*	41*	< 1	< 1	< 1	< 1	1*	< 1	1*	1.4e+09	2.8e+07	1.2e+09*	1.1e+08
Permethrin	< 1	< 1	< 1	3.3*	< 1*	< 1	< 1	< 1	< 1	8e+09	5.9e+06	2.3e+06	4e+08
Cypermethrin	< 1	< 1	< 1	< 1	< 1	< 1	< 1	< 1	< 1	4.8e+02	< 1	6.2e+02	< 1
Cl2CA	< 1	< 1	< 1	< 1	< 1	< 1	< 1	< 1	< 1	7.7e+04	2.6e+04	42	2.8e+03
3-PBA	< 1	< 1	< 1	< 1	< 1	< 1	< 1	< 1	< 1	9.5e+03	5.3e+02	< 1	1.9e+03
Fipronil	< 1	< 1	< 1	1*	< 1	< 1	2.5*	1.6e+03*	< 1	4.1e+15*	4.7e+10*	9.7e+12*	2.8e+14*
Fipronil-sulfone	< 1	< 1	< 1	< 1	< 1	< 1	< 1	< 1	< 1	19	< 1	6.9	< 1
2-IPP	< 1	< 1	1	< 1	< 1	< 1	< 1	< 1	< 1	7.3e+04	1.8e+10	2.4e+03	2.5e+03
Carbofuran-phenol	< 1	< 1	< 1	< 1	< 1	< 1	< 1	< 1	< 1	19	< 1	6.6	< 1
Diflufenican	< 1	< 1	< 1	< 1	< 1	< 1	< 1	< 1	< 1	2.5e+06	3.7e+08	7.6e+06	2.9e+05
Trifluralin	3.6e+05*	9.9e+07*	< 1	2.7e+15*	1e+02*	2e+02*	4.9e+05*	9.1e+07*	1.5e+02*	3e+13*	1.6e+10	5e+13*	2.3e+11*
Oxadiazon	< 1	< 1	< 1	< 1	< 1	< 1	< 1	< 1	< 1	1.3e+02	< 1	87	< 1

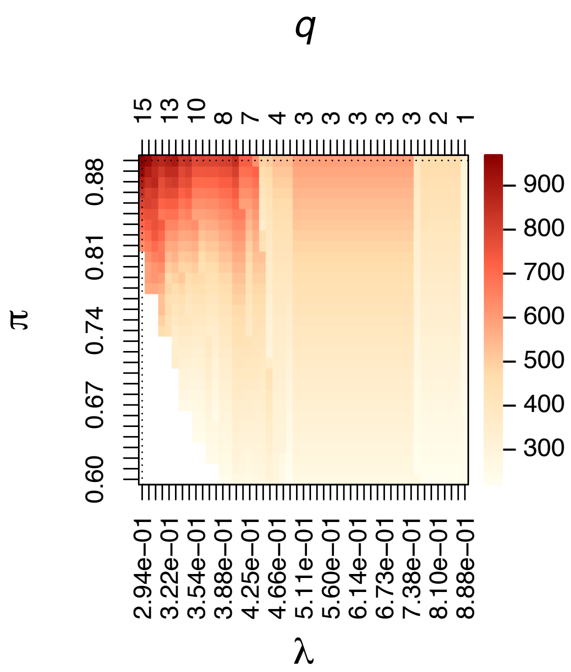

Figure S2: Calibration plot of conditional independence network of measured cardiometabolic traits. The stability score (colour-coded) for different penalty parameters λ and thresholds in selection proportion π. q = numbers of features selected. Calibration of the stability-enhanced graphical LASSO ensures that the expected number of False Positives (PFER) is below 10.

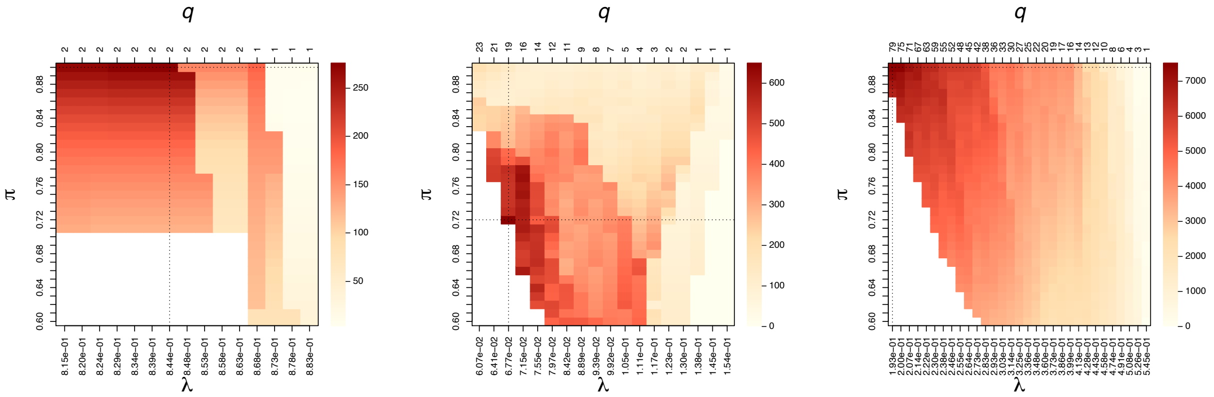

Figure S3: Block-specific calibration plots of conditional independence network of exposures (dietary intake and chemical measurements). The three blocks are calibrated separately: diet-diet (left), diet-chemical (middle) and chemical-chemical (right). The stability score (colour-coded) for different penalty parameters λ and thresholds in selection proportion π. q = numbers of features selected. Calibration of the stability-enhanced graphical LASSO ensures that the expected number of False Positives (PFER) is below 20.

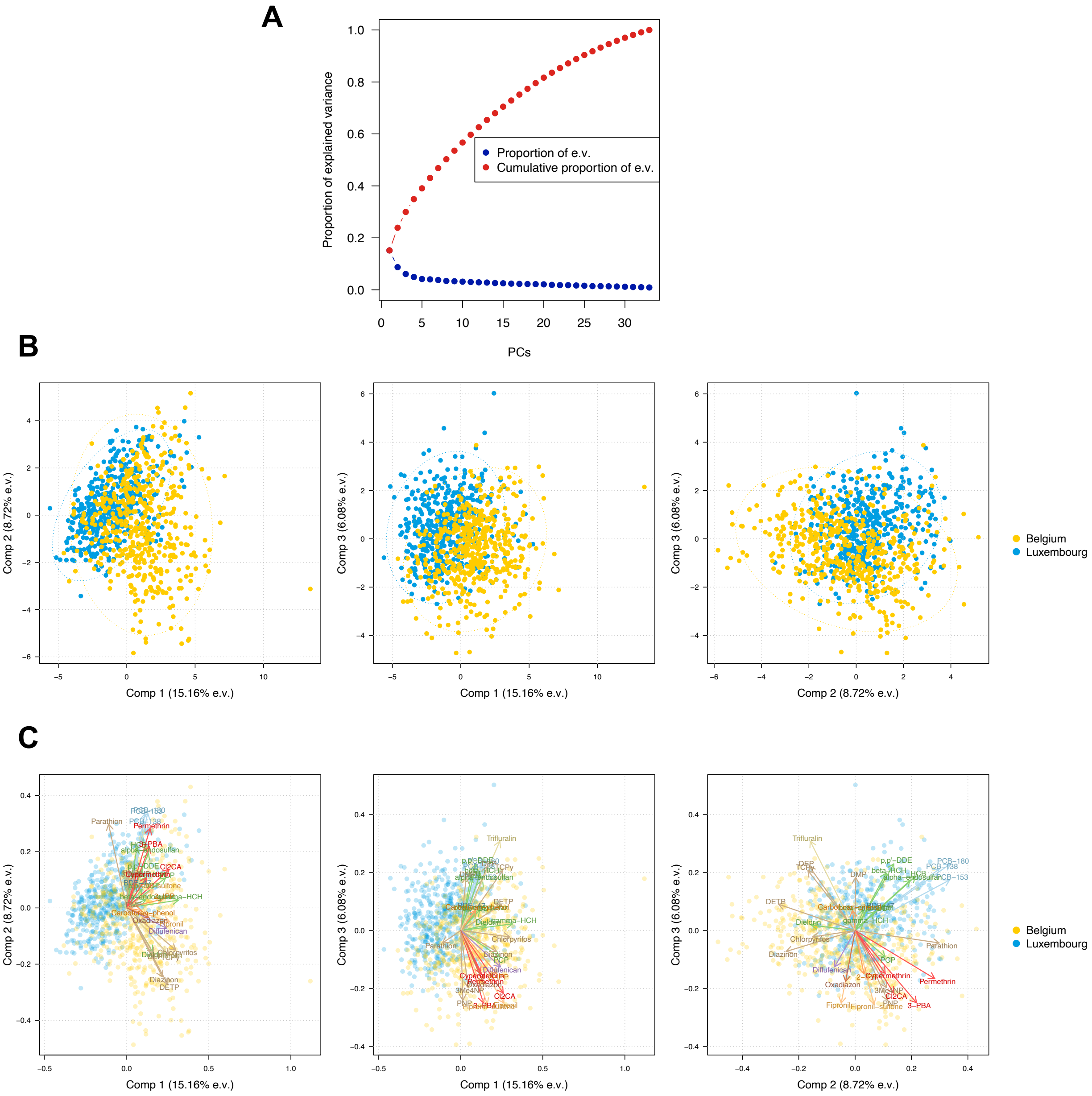

Figure S4: Results from the Principal Component Analysis (PCA) of the hair matrix of 941 participants. The (cumulative) proportion of explained variance (e.v.) of each principal component (PC) is shown (A). The projection of the participants in the first three components are represented as points coloured by assessment centre (B). The ellipse represents 95% pairwise confidence regions for each stratified group. The loading of each variable in the first three principal components is represented by a vector coloured by chemical family (C).

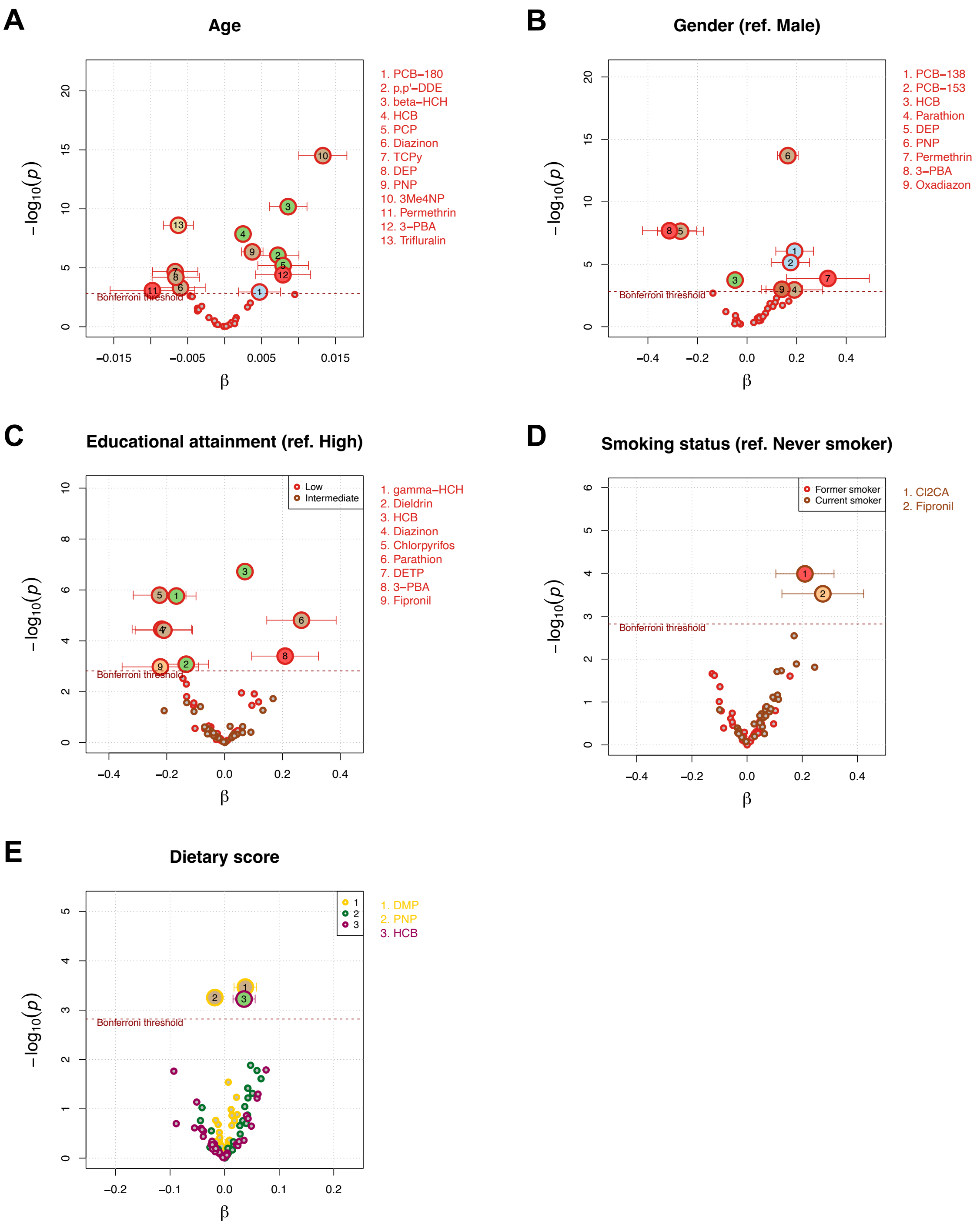

Figure S5: The marginal association between age (A), gender (B), educational attainment (C), smoking status (D), and dietary intake scores (E) and hair-derived measurements of pollutant exposure (p=33). Each point represents the strength ($-log_{10}(p)$, y-axis) and effect size (β, x-axis) of an association. The error bars represent the 95% confidence interval of β. Significant associations ($p < 0.05/33$) are numbered and listed.

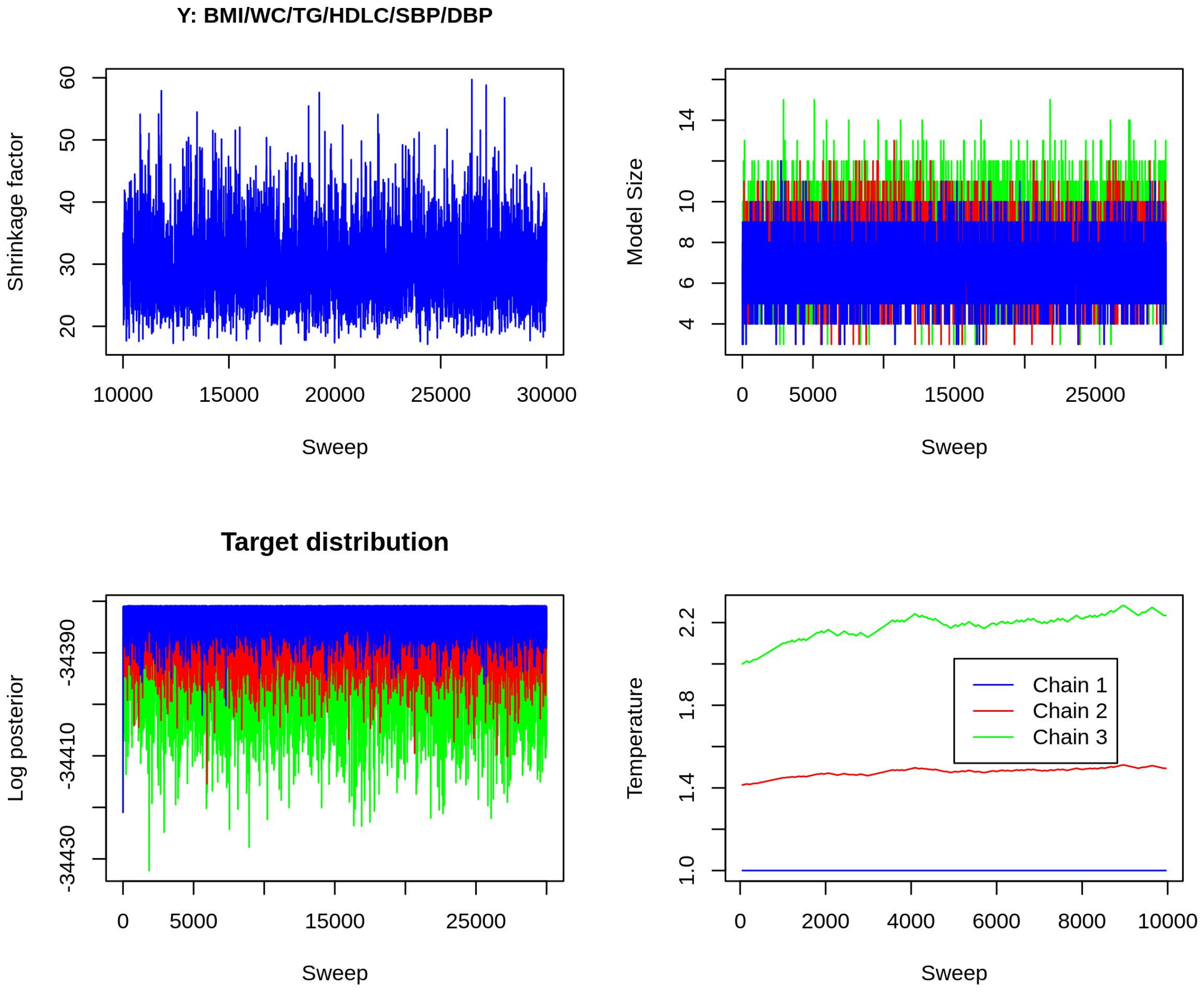

Figure S6: Example of plots showing convergence of GUESS algorithm in multi-trait analysis (with six traits as outcomes). The trace of the shrinkage factor (top left); the traces of the model sizes for the three different chains, showing, as expected, increased model sizes in heated chains (top right); the traces of the logarithm of the posterior distributions for the three different chains (bottom left); the traces of the temperatures of the three different chains during burn-in (bottom right).
